# Subclass and avidity of circumsporozoite protein specific antibodies associate with protection status against malaria infection

**DOI:** 10.1038/s41541-021-00372-x

**Published:** 2021-08-30

**Authors:** Kelly E. Seaton, Rachel L. Spreng, Milite Abraha, Matthew Reichartz, Michelle Rojas, Frederick Feely, Richard H. C. Huntwork, Sheetij Dutta, Sarah V. Mudrak, S. Munir Alam, Scott Gregory, Erik Jongert, Margherita Coccia, Fernando Ulloa-Montoya, Ulrike Wille-Reece, Georgia D. Tomaras, S. Moses Dennison

**Affiliations:** 1 Duke Human Vaccine Institute, Durham, NC USA; 2 Duke Center for Human Systems Immunology, Durham, NC USA; 3 Duke University Department of Surgery, Durham, NC USA; 4 Walter Reed Army Institute of Research, Silver Spring, MD USA; 5 Duke University Department of Pathology, Durham, NC USA; 6 PATH’s Malaria Vaccine Initiative, Washington, DC USA; 7 GSK, Rixensart, Belgium; 8 Duke University Department of Immunology, Durham, NC USA; 9 Duke University Department of Molecular Genetics and Microbiology, Durham, NC USA; 10 Present Address: GSK, Rockville, MD USA

**Keywords:** Infectious diseases, Malaria, Immunology

## Abstract

RTS,S/AS01 is an advanced pre-erythrocytic malaria vaccine candidate with demonstrated vaccine efficacy up to 86.7% in controlled human malaria infection (CHMI) studies; however, reproducible immune correlates of protection (CoP) are elusive. To identify candidates of humoral correlates of vaccine mediated protection, we measured antibody magnitude, subclass, and avidity for *Plasmodium falciparum* (Pf) circumsporozoite protein (CSP) by multiplex assays in two CHMI studies with varying RTS,S/AS01B vaccine dose and timing regimens. Central repeat (NANP6) IgG1 magnitude correlated best with protection status in univariate analyses and was the most predictive for protection in a multivariate model. NANP6 IgG3 magnitude, CSP IgG1 magnitude, and total serum antibody dissociation phase area-under-the-curve for NANP6, CSP, NPNA3, and N-interface binding were also associated with protection status in the regimen adjusted univariate analysis. Identification of multiple immune response features that associate with protection status, such as antibody subclasses, fine specificity and avidity reported here may accelerate development of highly efficacious vaccines against *P. falciparum*.

## Introduction

Malaria is a global disease with an impact of over 229 million cases and an estimated 409,000 deaths in 2019 worldwide^1^. Public health specialists and scientists have set forth the vision to reduce mortality and incidence by 90% compared to 2015 levels as part of the World Health Organization (WHO) global technical strategy for malaria 2016–2030^2^. A deeper understanding of what constitutes protective immunity will be instrumental in focusing prevention efforts toward achieving global targets for malaria reduction and elimination by the year 2030^2^.

Malaria disease is caused by parasites of the genus *Plasmodium (P.)* that can transmit from human to human through the bite of an infected Anopheles mosquito. Sporozoites are released into the bloodstream and target hepatocytes in the liver to initiate the parasitic life cycle in humans. The *P. falciparum* circumsporozoite protein (CSP) is necessary for adhesion and entry into human hepatocytes^3,4^ and is considered a leading target for protective antibodies. CSP is composed of a central repeat region (NANP and NVDP amino acid repeats) flanked on either side by two conserved regions, the N-terminal domain and the C terminal region with a glycophosphatidylinositol (GPI) anchor domain for attachment to the sporozoite membrane. The repeat region contains four NVDP and 38 NANP repeats (i.e. 3D7 reference strain) that can differ among strains^5^ and among adults and children^6^. Antibody responses to an epitope positioned between the N terminus and the central repeat domain of CSP was also recently identified as a key target for antibody mediated protection^7,8^.

The most advanced malaria vaccine is RTS,S, a *P. falciparum* CSP based vaccine consisting of 19 NANP repeats and the C-terminal CSP region fused to the Hepatitis B Surface antigen^9–11^ combined with the AS01 adjuvant system to elicit immunity against the sporozoite at the pre-erythrocytic stage of the parasite life cycle. The RTS,S vaccine was moderately efficacious in Phase 3 trials in Africa with 53.9% and 32.9% vaccine efficacy (VE) against clinical malaria in children and infants over the 14 months of initial follow-up^12–15^. The VE waned over time for both children and infants; for children, final VE after 48 months of follow-up was 36.3%, and for infants, final VE after 38 months of follow-up was 25.9%. In a Phase 2a controlled human malaria infection (CHMI) study in adults, modification of this vaccine regimen to include a delayed fractional third dose with AS01B adjuvant improved VE to 86.7% (95% CI, 66.8–94.6%) at 28 days after challenge^16^. To test whether the higher VE reported in CHMI trials translates to field settings for children, further testing is ongoing in malaria endemic regions (Clinicaltrials.gov identifier: NCT03276962).

Identification of antibody correlates/surrogates of protection for efficacious malaria vaccines will result in an efficient and cost-effective pathway toward product development and licensure by providing a benchmark for future vaccine development to improve VE and durability. Thus, delineating the anti-infective properties of vaccine-induced antibodies that correspond with protection is critical. High levels of vaccine-induced total IgG anti-CSP antibodies were associated with protection in clinical studies against clinical malaria disease^17–19^ and against Pf sporozoite infection^16,18,20^. However, antibody titers are not predictive in some cases and are not an established correlate of protection^16,21^. CSP specific antibodies are functional and can block infection in animal models^22,23^. Although two studies evaluating total IgG anti-CSP avidity to *P. falciparum* antigens by ELISA indicated that there is no association between avidity score and protection^24,25^, one study has suggested higher-avidity IgG4 antibodies may inhibit opsonophagocytosis and thereby help protect against infection^26^. FcγRIIIa engagement and phagocytosis were also shown to predict protection in the MAL-068 CHMI trial^27^. These data together with evidence from other vaccine correlates studies^28^ indicate that the roles of antibody isoforms, specificities and functional attributes in providing protecting immunity are complex and require further examination. Thus, deciphering the biophysical properties of antibodies that are modulated by antigen dosing, vaccine intervals and adjuvants is critical to understanding potential immune mechanisms of protection.

In this study, we characterized the humoral immune response in two Phase 2 CHMI studies to fully probe vaccine-elicited epitope and subclass specific responses, including binding antibody magnitude and avidity measures. We identified several antibody measurements that associated with protection status using univariate and multivariate analyses, and report down-selected candidate measurements for testing in future immune correlates of protection (CoP) studies, including currently ongoing field trials.

## Results

### Controlled Human Malaria Infection Model (CHMI)

For this study, we assessed two CHMI RTS,S/AS01B vaccine studies that demonstrated 44-86.7% vaccine efficacy^16,20^. In the first study (NCT01366534), referred to throughout as MAL-068, study participants received either one dose of Ad35.CS.01 vaccine followed by two doses of RTS,S/AS01 (ARR group) or three doses of RTS,S/AS01 (RRR group) at months 0, 1, and 2 followed by CHMI three weeks after the final vaccination^20^. In the second study (NCT01857869), referred to throughout as MAL-071, study participants received either a standard dose regimen of RTS,S/AS01 at months 0, 1, and 2 (RRR group) or two standard doses of RTS,S/AS01 at months 0 and 1 followed by a one fifth fractional third dose of RTS,S/AS01 at month 7 (RR_r group); both study arms underwent CHMI three weeks following last vaccination^16^. For both studies, we examined the specificity, magnitude and quality of the antibody response by measuring vaccinees’ serum antibody binding to: 1) full-length CSP, 2) the central repeat region peptides NANP6 (six NANP repeats) and NPNA3 (two NANP repeats, which has exhibited improved discrimination of low avidity antibodies^29^), 3) N-interface peptide with sequence corresponding to the N-terminal junction region of CSP, and 4) the CSP C-terminal region peptide PF16, a marker commonly used to assess anti-CSP responses^30^. Serum binding antibody responses and avidity measurements were measured by biolayer interferometry (BLI) and IgG subclass-specific binding antibody levels and avidity index (AI) were investigated by binding antibody multiplex assay (BAMA).

To harmonize identification of common humoral immune responses associated with protection status in RTS,S CHMI vaccine trials given the differences in vaccine type (ARR vs RRR) and regimen dose and schedule (RRR vs RR_r), we analyzed serum antibody responses elicited by each vaccine type as well as between trials on the day of challenge (DOC). We examined thirty-five epitope and subclass-specific binding antibody responses by BAMA (Tables 1 and 2 summarize the medians and 25^th^ and 75^th^ percentiles by study arm and protection status) and 15 different BLI measurements including specificity, magnitude and off rate (dissociation rate constant). Table 3 summarizes the medians and 25^th^ and 75^th^ percentiles by study arm and protection status, for BLI total serum measurements, and the individual antibody measurements by BAMA and BLI for both arms of MAL-068 are shown in Supplementary Figs. 1-5.

**Table 1:** Descriptive statistics for subclass antibody measures in RTS,S/AS01 vaccinees (measured in both studies).

Measurement	Protection Status	ARR	RRR	RR_r
**IgG1 CSP (MFI)**	Protected	1.9e7 (1.1e7, 3.1e7)	3.1e7 (1.9e7, 4.5e7)	1.6e7 (1.2e7, 3.2e7)
	Infected	9.3e6 (7.0e6, 2.3e7)	1.8e7 (1.4e7, 3.9e7)	1.9e7 (1.7e7, 2.1e7)
IgG2 CSP (MFI)	Protected	8.7e5 (5.9e5, 9.0e6)	5.3e5 (2.2e5, 9.1e5)	6.7e5 (4.4e5, 2.2e6)
	Infected	5.1e5 (1.1e5, 1.5e6)	7.9e5 (5.9e5, 2.3e6)	3.5e5 (3.0e5, 4.6e5)
IgG3 CSP (MFI)	Protected	8.9e5 (6.2e5, 4.7e6)	5.1e6 (1.0e6, 8.8e6)	6.4e5 (2.8e5, 2.1e6)
	Infected	4.0e6 (2.8e6, 4.5e6)	6.9e5 (5.8e5, 6.8e6)	7.2e5 (4.3e5, 1.0e6)
IgG4 CSP (MFI)	Protected	2.6e4 (1.8e4, 6.7e4)	5.1e4 (3.1e4, 9.9e4)	5.3e5 (1.5e5, 7.7e6)
	Infected	3.0e4 (2.1e4, 4.2e4)	7.0e4 (2.5e4, 1.9e5)	3.4e5 (9.6e4, 2.2e6)
**IgG1 NANP6 (MFI)**	Protected	8.4e4 (4.1e4, 2.3e5)	1.5e5 (1.3e5, 2.9e5)	2.1e5 (1.1e5, 4.7e5)
	Infected	2.7e4 (1.6e4, 5.1e4)	6.3e4 (1.1e4, 1.2e5)	2.0e5 (1.6e5, 2.7e5)
IgG2 NANP6 (MFI)	Protected	8.8e3 (5.0e3, 1.2e4)	2.7e4 (1.3e4, 4.0e4)	2.2e4 (1.0e4, 5.3e4)
	Infected	3.5e3 (1.5e3, 1.4e4)	1.8e4 (8.3e3, 9.0e4)	1.3e4 (1.1e4, 1.6e4)
**IgG3 NANP6 (MFI)**	Protected	2.1e4 (1.4e4, 2.3e4)	8.1e4 (3.8e4, 1.4e5)	2.5e4 (5.3e3, 6.0e4)
	Infected	1.1e4 (9.6e3, 5.3e4)	1.8e4 (6.1e3, 5.0e4)	2.6e4 (1.5e4, 4.7e4)
IgG4 NANP6 (MFI)	Protected	950.00 (693.75, 3.9e3)	1.4e3 (50.00, 3.3e3)	981.25 (50.00, 3.5e3)
	Infected	1.3e3 (484.38, 1.6e3)	1.1e3 (50.00, 1.9e3)	1.4e3 (325.00, 9.7e3)
IgG3 PF16 (MFI)	Protected	2.3e4 (1.7e4, 8.0e4)	2.8e4 (1.4e4, 1.8e5)	2.6e4 (9.1e3, 6.1e4)
	Infected	3.6e4 (2.3e4, 6.2e4)	4.3e4 (1.5e4, 9.7e4)	2.5e4 (1.1e4, 4.7e4)
IgG3 HepB (MFI)	Protected	6.8e3 (1.5e3, 1.1e5)	4.3e4 (1.9e4, 2.0e5)	1.0e5 (1.2e4, 3.5e5)
	Infected	3.5e4 (4.7e3, 9.7e4)	3.6e4 (5.4e3, 2.1e5)	1.1e5 (6.6e4, 2.1e5)
IgG1 CSP AI	Protected	63 (48.5, 75.5)	71 (63, 83)	87.5 (78, 92.25)
	Infected	64 (48, 73)	62 (43.5, 74.5)	93.5 (84.5, 94.25)
IgG2 CSP AI	Protected	47 (34.75, 83.75)	113.5 (85, 158.25)	87.5 (69, 103.25)
	Infected	56 (39.5, 72.25)	80 (58.75, 93.75)	70.5 (66.75, 74.5)
IgG3 CSP AI	Protected	75 (63, 82.5)	78 (70, 89)	97 (82, 101)
	Infected	75 (70, 84)	79.5 (67.75, 84)	84 (81.75, 87.75)
IgG4 CSP AI	Protected	61.5 (45.25, 71.75)	66 (54, 73)	75.5 (56.75, 84.5)
	Infected	71 (62, 81)	63 (57.5, 67)	72.5 (70.25, 78)
IgG1 NANP6 AI	Protected	50 (47, 62)	55 (43.75, 69.75)	82 (73, 89)
	Infected	57 (52, 63)	60.5 (56.75, 65)	69 (65.75, 71.75)
IgG2 NANP6 AI	Protected	47 (42, 57)	57.5 (45, 80.5)	76 (68, 93)
	Infected	33 (16, 56)	53 (44, 58)	65 (61.5, 76)
IgG3 NANP6 AI	Protected	69 (63, 79)	62 (52, 80)	74.5 (70.75, 81.5)
	Infected	67 (58, 72)	74 (70, 77)	40 (36.5, 64.5)

The median and 25^th^ to 75^th^ percentiles, or interquartile range (IQR) indicated in parentheses, of magnitude (MFI x Dilution Factor) and AI (%) are shown. RRR summary statistics represent the standard dose regimen groups from both studies combined. AI is reported only for positive responses. Top univariate predictors are in bold.

**Table 2:** Descriptive statistics for subclass measures in MAL-068 vaccinees which were not measured in MAL-071.

Measurement	Protection Status	ARR	RRR
IgG3 NPNA3 (MFI)	Protected	9.7e4 (4.6e4, 1.3e5)	4.2e5 (2.4e5, 6.5e5)
	Infected	5.4e4 (2.9e4, 2.2e5)	7.3e4 (3.0e4, 3.3e5)
IgG4 NPNA3 (MFI)	Protected	2.4e4 (1.1e4, 3.2e4)	2.7e4 (2.2e4, 3.4e4)
	Infected	3.2e4 (1.5e4, 6.9e4)	1.6e4 (7.5e3, 2.3e4)
IgG1 PF16 (MFI)	Protected	1.2e5 (1.1e5, 1.4e5)	2.0e5 (1.4e5, 2.8e5)
	Infected	1.5e5 (9.8e4, 1.9e5)	1.9e5 (1.3e5, 3.9e5)
IgG2 PF16 (MFI)	Protected	2.5e4 (6.9e3, 5.7e4)	6.3e4 (2.6e3, 1.4e5)
	Infected	1.5e4 (7.4e3, 3.6e4)	2.1e4 (1.3e4, 1.3e5)
IgG4 PF16 (MFI)	Protected	1.7e3 (650.00, 6.4e3)	6.7e3 (3.7e3, 2.9e4)
	Infected	3.9e3 (1.2e3, 7.7e3)	6.0e3 (3.0e3, 1.3e4)
IgG1 HepB (MFI)	Protected	1.0e5 (6.4e4, 1.4e5)	1.7e5 (8.3e4, 2.9e5)
	Infected	1.2e5 (1.1e4, 2.3e5)	2.1e5 (1.3e5, 4.1e5)
IgG2 HepB (MFI)	Protected	237.50 (12.50, 393.75)	50.00 (0.00, 121.88)
	Infected	181.25 (0.00, 321.88)	0.00 (0.00, 259.38)
IgG4 HepB (MFI)	Protected	575.00 (362.50, 1.7e3)	1.3e3 (793.75, 1.7e3)
	Infected	1.2e3 (125.00, 2.5e3)	3.0e3 (890.62, 5.7e3)
IgG3 NPNA3 AI	Protected	74.5 (63, 80)	71 (57.5, 78.5)
	Infected	73 (67.5, 80)	78 (73, 88)
IgG1 PF16 AI	Protected	31 (23.25, 40.25)	35.5 (25.5, 41)
	Infected	25 (22, 27)	33.5 (29.75, 41.5)
IgG3 PF16 AI	Protected	42.5 (33.25, 43.25)	36 (33, 44.5)
	Infected	34 (27.75, 45.25)	48 (41, 58)
IgG1 HepB AI	Protected	58 (43.5, 77.75)	45 (32.25, 55.25)
	Infected	80 (61, 86)	73 (62.5, 81.5)
IgG3 HepB AI	Protected	68 (68, 68)	24 (13.5, 34.5)
	Infected	75 (68, 79)	60 (60, 63.5)

The median and 25^th^ to 75^th^ percentiles, or interquartile range (IQR) indicated in parentheses, of magnitude (MFI x Dilution Factor) and AI (%) are shown. AI is reported only for positive responses.

**Table 3:** Descriptive statistics for serum measures in RTS,S/AS01 vaccinees from MAL-068 and MAL-071 studies.

Measurement	Protection Status	ARR	RRR	RR_r
CSP (nm)	Protected	0.55 (0.50, 0.86)	1.25 (1.05, 1.54)	0.79 (0.67, 1.41)
	Infected	0.34 (0.19, 0.50)	1.12 (0.37, 1.28)	0.83 (0.67, 0.95)
NANP6 (nm)	Protected	0.57 (0.45, 0.71)	1.18 (0.99, 1.59)	0.92 (0.68, 1.26)
	Infected	0.37 (0.18, 0.52)	0.82 (0.49, 0.99)	0.69 (0.61, 0.89)
NPNA3 (nm)	Protected	0.25 (0.24, 0.48)	0.57 (0.34, 0.78)	0.31 (0.23, 0.39)
	Infected	0.21 (0.14, 0.29)	0.36 (0.21, 0.57)	0.19 (0.16, 0.24)
N-interface (nm)	Protected	0.08 (0.05, 0.17)	0.16 (0.10, 0.23)	0.11 (0.06, 0.20)
	Infected	0.06 (0.04, 0.08)	0.07 (0.03, 0.21)	0.06 (0.04, 0.07)
PF16 (nm)	Protected	0.16 (0.01, 0.18)	0.16 (0.09, 0.31)	0.20 (0.11, 0.29)
	Infected	0.09 (0.03, 0.13)	0.14 (0.10, 0.31)	0.19 (0.15, 0.20)
CSP off rate (s^−1^)	Protected	8.1e-4 (3.2e-4, 1.0e-2)	2.6e-4 (2.3e-4, 3.1e-4)	1.3e-4 (7.3e-5, 2.4e-4)
	Infected	1.0e-2 (1.0e-2, 1.0e-2)	2.6e-4 (1.8e-4, 1.0e-2)	3.0e-4 (2.2e-4, 2.8e-3)
NANP6 off rate (s^−1^)	Protected	1.0e-2 (3.7e-4, 1.0e-2)	2.2e-4 (1.6e-4, 2.8e-4)	3.0e-4 (2.2e-4, 4.8e-4)
	Infected	1.0e-2 (1.0e-2, 1.0e-2)	3.2e-4 (1.6e-4, 7.6e-3)	4.4e-4 (3.6e-4, 2.9e-3)
NPNA3 off rate (s^−1^)	Protected	1.0e-2 (7.1e-4, 1.0e-2)	7.5e-4 (4.6e-4, 3.4e-3)	1.0e-2 (4.8e-4, 1.0e-2)
	Infected	1.0e-2 (7.7e-3, 1.0e-2)	7.2e-4 (4.2e-4, 1.0e-2)	1.0e-2 (1.0e-2, 1.0e-2)
N-interface off rate (s^−1^)	Protected	8.0e-3 (1.1e-3, 1.0e-2)	1.6e-3 (9.5e-4, 1.0e-2)	1.7e-3 (1.4e-3, 1.0e-2)
	Infected	1.0e-2 (1.0e-2, 1.0e-2)	1.2e-3 (9.9e-4, 4.1e-3)	1.0e-2 (1.0e-2, 1.0e-2)
PF16 off rate (s^−1^)	Protected	1.0e-2 (2.9e-3, 1.0e-2)	4.9e-4 (4.2e-4, 1.0e-2)	6.2e-4 (2.7e-4, 1.0e-2)
	Infected	1.0e-2 (7.7e-3, 1.0e-2)	8.4e-4 (3.3e-4, 1.0e-2)	1.0e-2 (7.6e-3, 1.0e-2)
**CSP AUC**_**diss**_**(nm** **×** **s)**	Protected	165.02 (141.36, 253.14)	370.24 (318.37, 445.50)	237.24 (202.62, 427.82)
	Infected	99.44 (56.36, 142.88)	330.32 (106.57, 376.70)	244.41 (194.06, 281.17)
**NANP6 AUC**_**diss**_**(nm** **×** **s)**	Protected	160.65 (126.53, 195.56)	352.92 (282.57, 463.44)	261.04 (199.95, 373.24)
	Infected	96.93 (47.95, 140.66)	235.31 (136.70, 293.87)	196.84 (172.16, 250.48)
**NPNA3 AUC**_**diss**_**(nm** **×** **s)**	Protected	69.41 (62.11, 133.37)	150.16 (90.32, 227.30)	85.01 (59.23, 109.98)
	Infected	54.19 (37.44, 80.97)	94.02 (51.50, 154.60)	47.70 (37.01, 63.02)
**N-interface AUC**_**diss**_**(nm** **×** **s**)	Protected	14.94 (8.78, 32.36)	38.94 (16.05, 53.80)	24.00 (9.72, 43.65)
	Infected	10.85 (7.40, 15.39)	11.75 (2.24, 48.07)	10.85 (7.26, 14.41)
PF16 AUC_diss_ (nm × s)	Protected	41.06 (1.00, 46.38)	41.58 (20.42, 87.92)	56.08 (25.74, 79.66)
	Infected	23.36 (3.11, 32.89)	35.69 (24.08, 89.13)	51.30 (38.11, 56.75)

The median and 25^th^ to 75^th^ percentiles, or interquartile range (IQR) indicated in parentheses, of magnitude (nm), off rate (s^-1^), and AUC_diss_ (nm × s) are shown. RRR summary statistics represent the standard dose regimen groups from both studies combined. Off rate is reported only for positive responders, and was set to 1.0e-2 for positive responses less than the LLOQ. Top univariate predictors are in bold (only AUC_diss_ measurements were included in statistical analyses).

### Distinct vaccine elicited antibody responses

**Fig. 1:**
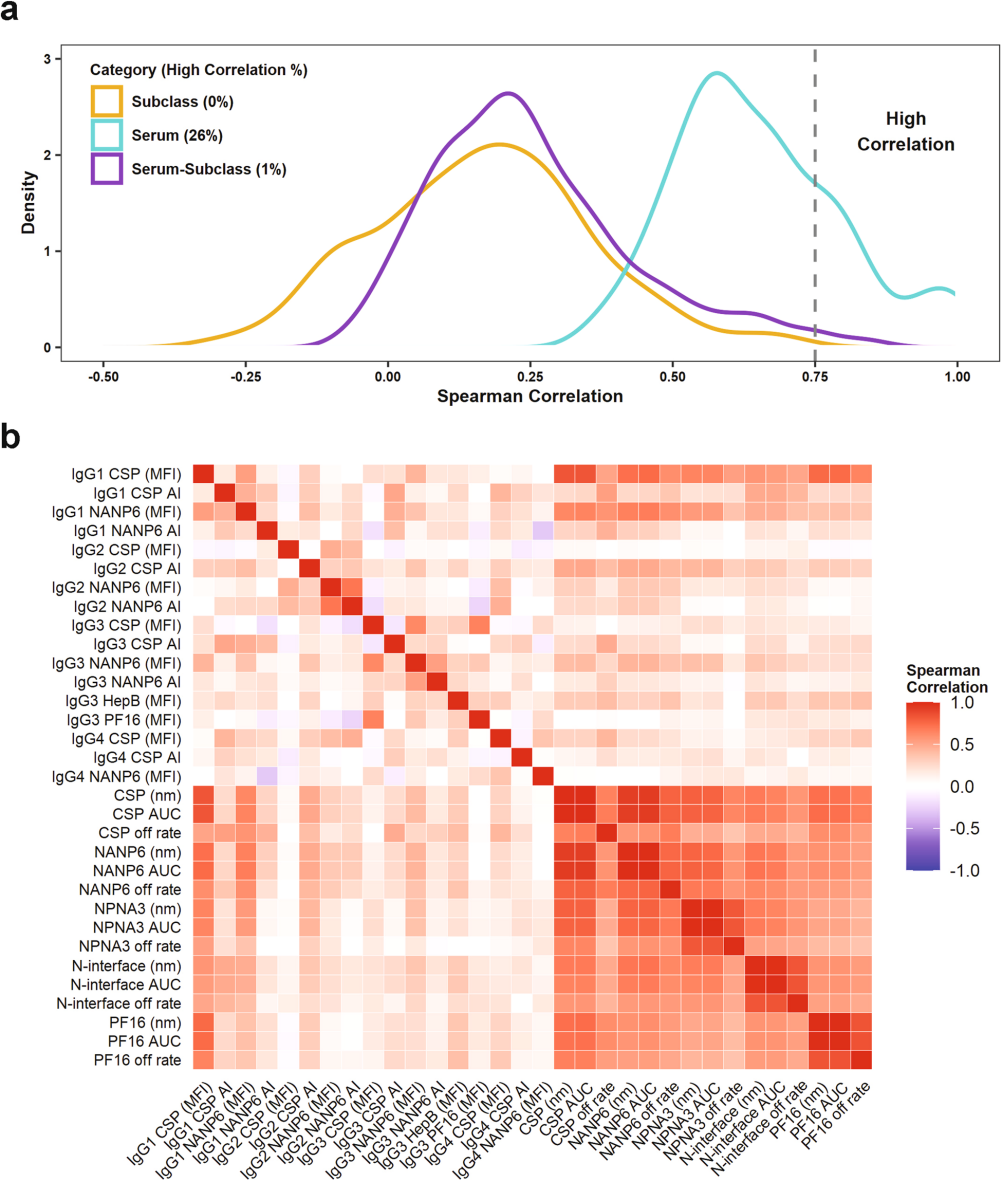
Distinct vaccine elicited antibody responses. The distribution of Spearman’s rank correlation coefficients is shown between measurements within and between assay types, with the percent of correlation coefficients above 0.75 indicated (**a**). A heatmap of all correlation values, is also shown. This heatmap is symmetric about the diagonal, with inter-assay subclass-serum correlations shown in the upper right and lower left quadrants, intra-assay subclass correlations shown in the upper left quadrant, and intra-assay serum correlations shown in the lower right quadrant (**b**).

To identify antibody measurements that represent distinct immune responses to vaccination, the correlation among antibody measurements within each trial for the DOC sera of vaccinees were first determined by Spearman’s rank correlation (Fig. 1 and Supplementary Tables 1–3). Among the subclass-specific antibody binding measurements (intra-assay correlations in the upper left quadrant of Fig. 1b and in Supplementary Table 1), there were no correlations above 0.75. Subclass-matched CSP specific binding magnitude and AI measurements had low correlation (*r* = -0.16 to *r* = 0.17), while subclass-matched NANP6 specific binding magnitude and AI measurements had low to moderate correlation (*r* = 0.25 for IgG1, *r* = 0.71 for IgG2, and *r* = 0.54 for IgG3). Note that IgG2 PF16 and HepB AI and IgG4 NANP6, NPNA3, PF16, and HepB AI were not included in correlation calculations or any of the following analyses since response rates were low (as can be seen for MAL-068 in Supplementary Fig. 1), leading to reportable AI values for few participants. CSP and NANP6 binding magnitudes had low to moderate correlation within subclass (*r* = 0.54 for IgG1, *r* = 0.47 for IgG2, *r* = 0.63 for IgG3, and *r* = 0.37 for IgG4), consistent with their unique immune function properties.

As expected, a fraction of the serum antibody measurements (26%) (intra-assay correlations in the lower right quadrant of Fig. 1b and in Table S2) were highly correlated (Fig. 1a). The area under the dissociation phase binding response curve (AUC_diss_) is a measurement which captures both magnitude and off rate and will be highly correlated with magnitude in cases where variability in off rates is low or if the magnitude is high with weak avidity. However, these variables will not be redundant within study arms with heterogeneity in both magnitude and off rate measurements. In these two studies, Spearman correlation coefficients were greater than 0.98 for epitope-matched AUC_diss_ and magnitude measurements, so we performed statistical analyses on AUC_diss_ only. Correlations across antigens were moderate to high for total serum BLI measurements (*r* = 0.51 to *r* = 0.94). CSP AUC_diss_ was highly correlated to both NANP6 AUC_diss_ and NPNA3 AUC_diss_ (*r* = 0.94 and *r* = 0.78, respectively).

In these studies, there were a few high correlations between subclass and serum measurements (inter-assay correlations shown in the upper right and lower left quadrants of Fig. 1b and in Supplementary Table 3, labeled as Serum-Subclass in Fig. 1a). The BLI serum binding responses (in nm) to different antigens were most correlated with IgG1 BAMA measurements compared to other subclass-specific responses (Fig. 1b). Serum CSP magnitude was highly correlated to IgG1 CSP (Spearman *r* = 0.84), but it showed little correlation to other subclasses (*r* = 0.08, *r* = 0.13, and r = 0.29 for IgG2, IgG3, and IgG4, respectively). Serum NANP6, NPNA3, N-interface, and PF16 magnitudes were also moderately to highly correlated with IgG1 CSP (*r* = 0.74, *r* = 0.65, *r* = 0.58, and *r* = 0.75, respectively). Serum NANP6 magnitude was moderately correlated to both IgG1 and IgG3 NANP6 (*r* = 0.67 and *r* = 0.44, respectively), but showed less correlation to IgG2 (*r* = 0.35) and no correlation to IgG4 (*r* = 0.00). Correlations between antigen-matched serum off rates and subclass-specific AI measurements were low to moderate (*r* = 0.17 to *r* = 0.53).

### NANP repeat specificities, antibody subclass and avidity measurements associate with protection against malaria infection

**Fig. 2:**
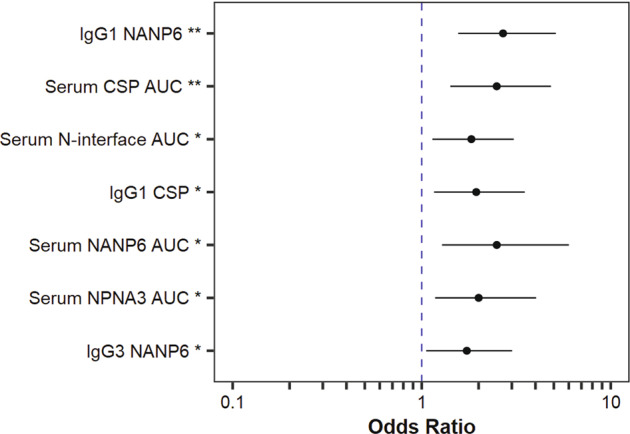
NANP repeat specificities, antibody subclass and avidity measurements associate with protection status. Odds ratios (circles) and 95% confidence intervals (error bars) obtained from logistic regression models fit individually to each immune measurement. Regression models were fit using both studies combined and were adjusted for regimen arm (Protection ~ Regimen + Measurement). Effects were considered significant and measurements are shown here if raw *p*-value < 0.05 and FDR-adjusted *p*-value < 0.2 (**p < 0.005 and FDR-adjusted *p* < 0.05, * *p* < 0.05 and FDR-adjusted *p* < 0.2).

We examined specificity, antibody form and avidity for associations with protection status and performed logistic regression analysis for each immune measurement individually. To increase statistical power, this analysis was performed on both studies combined, with the models adjusted for regimen. We identified seven univariate predictors of protection status with odds ratios (ORs) > 1 and 95% confidence intervals (CIs) that remain above OR = 1 (Fig. 2). Each of these immune measurements was statistically significant with *p*-value < 0.2 after false discovery rate (FDR)-adjustment and *p*-value < 0.05 before FDR-adjustment, with full results included in Table 4 for all measurements analyzed.

**Table 4:** Univariate Logistic Regression Results.

Measurement	OR (95% CI)	*p*-value	FDR-adjusted *p*-value
IgG1 CSP (MFI)	1.94 (1.16, 3.51)	0.017	0.091
IgG2 CSP (MFI)	1.23 (0.79, 2.03)	0.369	0.625
IgG3 CSP (MFI)	1.19 (0.73, 1.97)	0.495	0.769
IgG4 CSP (MFI)	1.37 (0.76, 2.62)	0.313	0.573
IgG1 NANP6 (MFI)	2.69 (1.56, 5.13)	0.001	0.021
IgG2 NANP6 (MFI)	1.16 (0.70, 1.93)	0.554	0.769
IgG3 NANP6 (MFI)	1.73 (1.06, 3.01)	0.037	0.117
IgG4 NANP6 (MFI)	1.16 (0.71, 1.93)	0.559	0.769
IgG3 PF16 (MFI)	1.11 (0.70, 1.77)	0.646	0.836
IgG3 HepB (MFI)	0.91 (0.57, 1.45)	0.697	0.852
IgG1 CSP AI	1.71 (0.96, 3.64)	0.110	0.303
IgG2 CSP AI	0.97 (0.58, 1.62)	0.896	0.940
IgG3 CSP AI	1.01 (0.61, 1.63)	0.968	0.968
IgG4 CSP AI	0.73 (0.42, 1.20)	0.234	0.467
IgG1 NANP6 AI	0.60 (0.26, 1.09)	0.145	0.319
IgG2 NANP6 AI	1.65 (0.87, 3.33)	0.129	0.315
IgG3 NANP6 AI	0.97 (0.54, 1.60)	0.898	0.940
CSP AUC_diss_ (nm × s)	2.49 (1.42, 4.83)	0.003	0.034
NANP6 AUC_diss_ (nm × s)	2.50 (1.28, 6.01)	0.021	0.091
NPNA3 AUC_diss_ (nm × s)	2.00 (1.18, 4.03)	0.025	0.091
N-interface AUC_diss_ (nm × s)	1.83 (1.14, 3.07)	0.016	0.091
PF16 AUC_diss_ (nm × s)	1.05 (0.65, 1.67)	0.838	0.940

For each univariate logistic regression model, the odds ratio (OR) with 95% confidence interval (CI) indicated in parentheses, *p*-value, and FDR-adjusted *p*-value are shown.

**Fig. 3:**
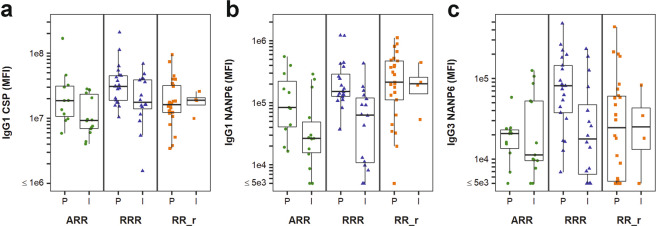
Three IgG antibody subclass measurements individually associate with protection from sporozoite infection in RTS,S/AS01 studies. Antibody responses on the day of challenge (DOC) were compared for the protected (P) and infected (I) groups of RTS,S/AS01 vaccinees from the Ad35.CS.01 prime (ARR, green circles), standard dose (RRR, blue triangles), and delayed fractional dose (RR_r, orange squares) regimens from two different Phase 2a studies. The top antibody subclass measurements identified were CSP specific IgG1 binding responses (**a**, *p* = 0.017 and FDR-adjusted *p* = 0.091), NANP6 specific IgG1 binding responses (**b**, *p* = 0.001 and FDR-adjusted *p* = 0.021), and NANP6 specific IgG3 binding responses (**c**, *p* = 0.037 and FDR-adjusted *p* = 0.117). *N* = 25 for MAL-068 ARR, *N* = 21 for MAL-068 RRR, *N* = 16 for MAL-071 RRR, and *N* = 30 for MAL-071 RR_r. The lower and upper hinges of the boxplots correspond to the 25th and 75th percentiles, with a line at the median. The lower and upper whisker extends from the box hinges to the smallest and largest values, respectively, which are within 1.5 * IQR of the hinge (where IQR, the inter-quartile range, is equal to the distance between the 25th and 75th percentiles).

Three of the seven predictors were subclass-specific measurements that were significantly associated with protection status in univariate analyses, including binding antibody levels for both NANP6 repeat and full-length CSP protein (Fig. 3). Here we also examined the effect size differences between the protected and infected groups. Protected RRR and ARR vaccinees had 1.7 and 2-fold higher median IgG1 magnitude to CSP, respectively (Fig. 3a and Table 1), with an OR of 1.94 (95% CI (1.16, 3.51), *p* = 0.017 and FDR-adjusted *p* = 0.091, Fig. 2 and Table 4). Protected RRR and ARR vaccinees had 2.4 and 3.1-fold higher median IgG1 magnitude to NANP6 (Fig. 3b and Table 1), respectively, with an OR of 2.69 (95% CI (1.56, 5.13), *p* = 0.001 and FDR-adjusted *p* = 0.021, Fig. 2 and Table 4). IgG3 NANP6 magnitude was 4.5-fold higher in RRR vaccinees and 1.8-fold higher in ARR vaccinees (Fig. 3c and Table 1), with an OR of 1.73 (95% CI (1.06, 3.01), *p* = 0.037 and FDR *p* = 0.117, Fig. 2 and Table 4). Median response differences for IgG1 CSP, IgG1 NANP6 and IgG3 NANP6 measurements were smaller between protected and infected RR_r vaccinees (fold differences in medians < 1.2).

**Fig. 4:**
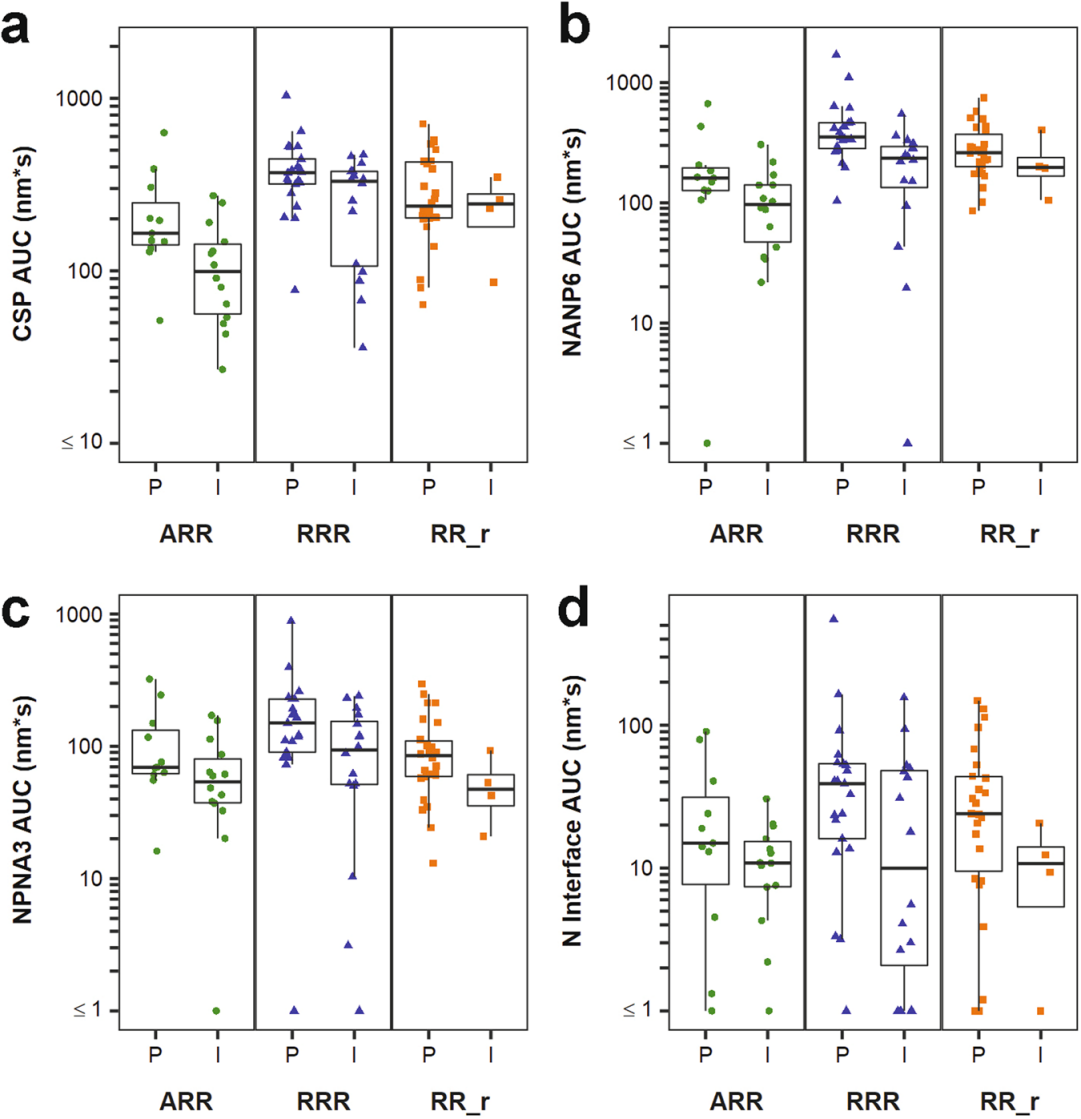
Four total serum antibody measurements individually associate with protection from sporozoite infection across RTS,S/AS01 studies. Antibody responses were compared on the day of challenge (DOC) for the protected (P) and infected (I) groups of RTS,S/AS01 vaccinees from the Ad35.CS.01 prime (ARR, green circles), standard dose (RRR, blue triangles), and delayed fractional dose (RR_r, orange squares) regimens from two different Phase 2a studies. The top total serum antibody measurements identified were CSP (**a**, *p* = 0.003 and FDR-adjusted *p* = 0.034), NANP6 (**b** *p* = 0.021 and FDR-adjusted *p* = 0.091), NPNA3 (**c**, *p* = 0.025 and *p*-adjusted = 0.091), and N-interface (**d**, *p* = 0.016 and FDR-adjusted *p* = 0.091) specific serum antibody AUC_diss_. *N* = 25 for MAL-068 ARR, *N* = 21 for MAL-068 RRR, *N* = 16 for MAL-071 RRR, and *N* = 30 for MAL-071 RR_r. Data shown for MAL-071 were previously reported and are displayed here in combination with MAL-068^32^. The lower and upper hinges of the boxplots correspond to the 25th and 75th percentiles, with a line at the median. The lower and upper whisker extends from the box hinges to the smallest and largest values, respectively, which are within 1.5 * IQR of the hinge (where IQR, the inter-quartile range, is equal to the distance between the 25th and 75th percentiles).

Four of the seven predictors were total serum antibody measurements, as measured by BLI, that associated with protection across the study arms (Fig. 4). CSP AUC_diss_ had an OR of 2.49 (95% CI (1.42, 4.83), *p* = 0.003 and FDR *p* = 0.034, Fig. 2 and Table 4) and was 1.7 fold higher in ARR protected vaccinees, but the medians were similar for protected and infected RRR and RR_r vaccinees (Fig. 4a and Table 3). Protected vaccinees had 1.3 to 1.7 fold higher NANP6 AUC_diss_ (Fig. 4b and Table 3), with OR = 2.50 (95%CI (1.28, 6.01), *p* = 0.021 and FDR *p* = 0.091, Fig. 2 and Table 4), and 1.3 to 1.8 fold higher NPNA3 AUC_diss_ (Fig. 4c and Table 3), with OR = 2.00 (95%CI (1.18, 4.03), *p* = 0.025 and FDR *p* = 0.091, Fig. 2 and Table 4). We also examined antibody responses to the N-interface region since some NANP6 repeat antibodies are cross-reactive^8,31^. As we previously reported for MAL-071, the median serum N-interface AUC_diss_ value was 6.4-fold higher in the protected vaccinees compared to the infected vaccinees in the MAL-071 RRR regimen^32^ and shown here in this combined analysis with MAL-068 measurements was 1.4, 3.3, and 2.2-fold higher in ARR, RRR, and RR_r, respectively (Fig. 4d and Table 3), with an OR of 1.83 (95%CI (1.14, 3.07), *p* = 0.016 and FDR *p* = 0.091 Fig. 2 and Table 4). As stated above, based on high correlations within BLI measurements, only AUC_diss_ was included in statistical analyses for each antigen. While BLI response magnitudes and off rates were not formally tested for associations with protection in the work presented here, as previously published^32^ and as seen in Supplementary Figs. 3-5, the association of AUC_diss_ measurements with protection in these two studies largely reflects the trends observed for magnitude measurements, with little variation seen among measurable off rates between regimen arms.

We identified four antibody measurements that were higher in the protected vs. infected vaccinees across each of the vaccine regimens, indicating potential common correlates of protection (i.e. CSP, NANP6, NPNA3, and N-interface AUC_diss_). For some of these measurements, differences were subtle, and follow-up analyses in larger cohorts will be necessary to determine whether identification of a protective antibody avidity threshold is possible. We also identified several measurements that were higher in protected vaccinees only within certain arms. In addition to the measurements discussed above which were significantly different in logistic regression models across studies adjusted for regimen (IgG1 CSP, IgG1 NANP6, and IgG3 NANP6), RRR protected vaccinees had 7.3-fold higher IgG3 CSP (Table 1) and 3-fold higher IgG2 PF16 (Table 2). The differences in trends with protection status observed within each regimen for these univariate immune correlates reflect heterogeneity among the vaccine arms and could suggest multiple underlying immunological pathways to protective immunity.

### Multivariate prediction of protection status

Immune correlates of protection can be complex^28,33^ and involve multiple measurements of the immune response. We hypothesized that a combination of antibody measurements would associate with protection status better than a single measurement since multiple antibody forms and specificities may best represent the immune mechanisms needed to prevent sporozoite invasion in vivo. To build a multivariate predictive model of protection for the RTS,S/AS01 vaccinees, we applied Least Absolute Shrinkage and Selection Operator (LASSO) penalized logistic regression. In addition to the pairwise Spearman rank correlation coefficients shown in Fig. 1, we also used variance inflation factor (VIF) to examine multicollinearity in the full logistic regression model containing the 22 different antibody measurements for both MAL-068 and MAL-071. CSP AUC_diss_ was the only measurement with a VIF greater than 10 (VIF = 14.3) and was excluded from the LASSO penalized regression. With CSP AUC_diss_ removed from the full model, the measurements with the largest VIF scores were IgG1 CSP, IgG3 CSP, and IgG3 NANP6 binding magnitude (VIF = 5.0, 5.1, and 5.1, respectively). These were retained for the remaining analysis, leaving 21 antibody measurements and 2 regimen indicator variables (0 or 1 values indicating RR_r or ARR immunization, where 1 = yes, and a 0 for both implies RRR immunization).

**Fig. 5:**
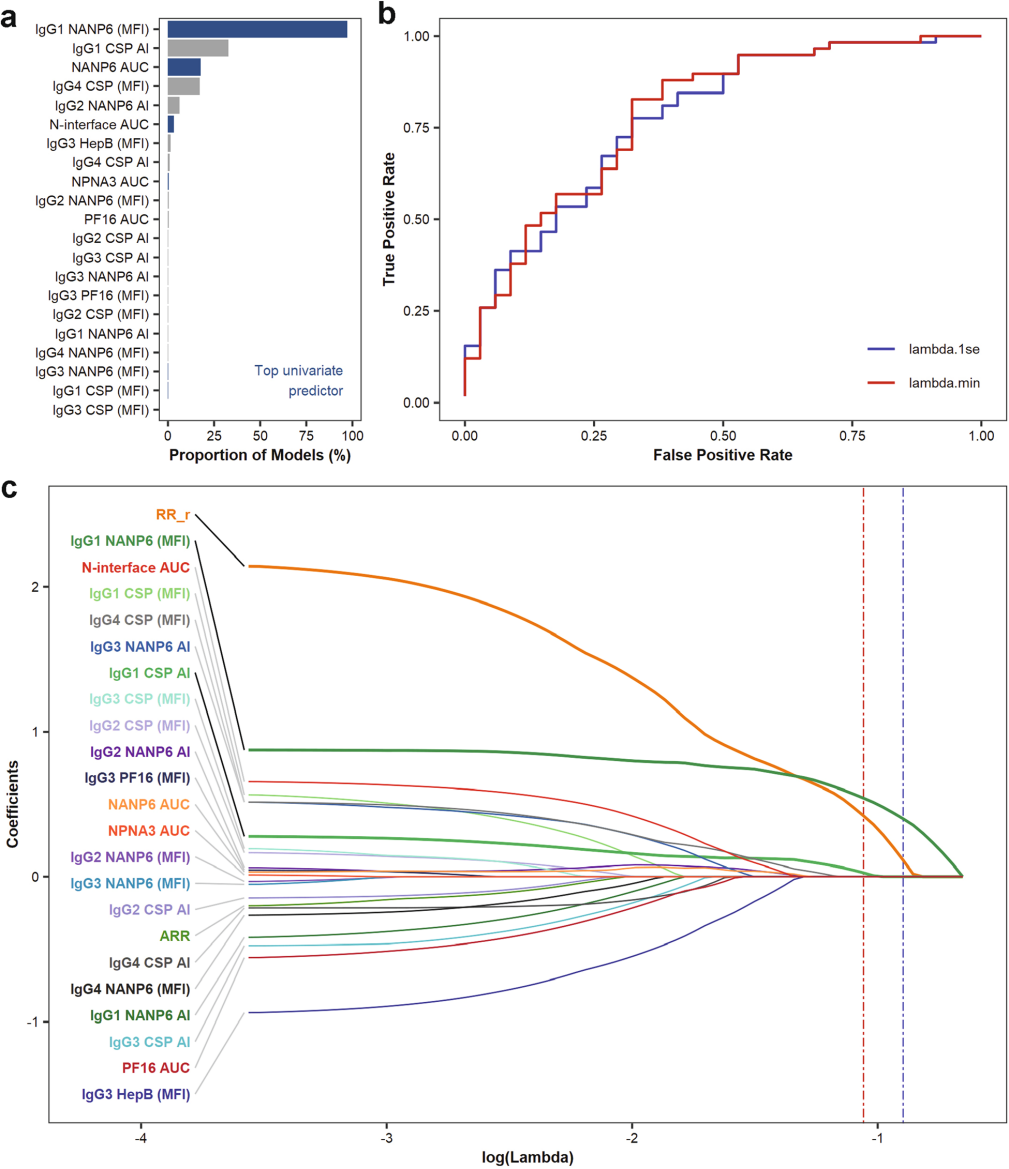
Frequency of putative protective immune measurements in computational models. Immune measurements are ranked by the proportion of cross-validation LASSO penalized models in which they appear (**a**). Blue bars represent measurements which were found to be associated with protection by univariate analyses. The most frequent measurements, IgG1 NANP6 magnitude and IgG1 CSP AI, also appear in the LASSO penalized model with λ equal to lambda.min, the value of the tuning parameter corresponding to the minimum cross-validation misclassification error. IgG1 NANP6 magnitude appeared in the LASSO penalized model with λ equal to lambda.1se, the largest value of λ such that misclassification error was within 1 standard error of the minimum. The ROC curves for these models are shown (**b**) and had AUC values of 0.788 (λ = lambda.min, red curve) and 0.779 (λ = lambda.1se, blue curve). The coefficients for the included measurements are shown along the entire path of λ values (**c**), with lambda.min and lambda.1se indicated by the vertical red and blue lines, respectively. Each colored line corresponds to either a regimen indicator, RR_r or ARR (0 or 1 value, where 1 = yes), or an immune measurement. Variables included in the model with λ = lambda.min are represented by thicker lines. Smaller values of λ correspond to less penalization and will result in more measurements included in the regression model.

First, 1000 rounds of 5-fold cross-validated LASSO penalized regression was performed to characterize the stability of the regression models. IgG1 NANP6 binding magnitude was included in 97% of the cross-validation models and was the only measure to always have a positive coefficient when included (Fig. 5a). IgG1 CSP AI, NANP6 AUC_diss_, and IgG4 CSP binding magnitude were the only other measurements to appear in at least 10% of the cross-validation models. Next, LASSO penalized regression was performed on the entire data set combined. Receiver operating characteristic (ROC) curves for the models with the tuning parameter λ equal to that which minimized leave-one-out cross-validation misclassification (lambda.min) and the largest value of λ such that misclassification error was within 1 standard error of the minimum (lambda.1se) had AUC values of 0.788 and 0.779, respectively (Fig. 5b). The ROC curves are calculated on the training set and these AUC values may be an over-estimate, so future studies in field trials^12–15^ can serve as an independent validation set. The trace plot shows the coefficients of each measurement along the entire path of λ values (Fig. 5c), where smaller λ values correspond to less penalization and more variables in the regression model. The model with λ equal to lambda.min, indicated by the vertical red line, included an indicator variable for the RR_r regimen (shown as thick orange line), IgG1 NANP6 binding magnitude (thick dark green line), and IgG1 CSP AI (thick light green line). IgG1 CSP AI had a smaller contribution and was not included in the model with λ equal to lambda.1se, indicated by the vertical blue line. While the inclusion of IgG1 NANP6 binding magnitude is consistent with univariate analyses, the cross-validation classification errors of 27.2–31.5% and the ROC AUCs of 0.788–0.779 could suggest that there are other contributing factors not captured by the immune measurements included here. Although increased IgG1 NANP6 binding is associated with protection across regimen arms (Fig. 3b), the large amount of heterogeneity within and between study arms make the identification of a protective threshold difficult. Protected and infected RR_r vaccinees had similar median IgG1 NANP6 binding, but those medians were similar to slightly higher compared to protected ARR and RRR vaccinees. All regimen arms across MAL-068 and MAL-071 were included in analyses to maximize statistical power and to produce the most generalizable model, but the high efficacy of the MAL-071 RR_r regimen is a limitation. Since efficacy was higher in the RR_r arm compared to RRR and ARR, immunization with the RR_r regimen is a relatively good predictor of protection even in the absence of immunological data, which explains its large contribution to the LASSO penalized models (Fig. 5c). Along with the univariate measurements found to be associated with protection (Figs. 3 and 4), the top-ranking immune measurements that came up commonly across cross-validation models (Fig. 5a) generate hypotheses for further testing these measurements in independent clinical trials. Follow-up analyses on immunological data from trials with a larger number of participants will be important to validate and refine these results.

## Discussion

The RTS,S/AS01 controlled human malaria infection studies enable rapid interrogation of potential immune correlates of protection. Although the mechanisms responsible for protective immunity against malaria are unknown, antibodies that prevent liver-stage infection and red blood cell infection likely play a key role in protection. There is a critical need for improved malaria vaccines and bridging of effective strategies to diverse populations. The identification of a reproducible correlate of protection would rapidly accelerate this vaccine development. Given the complexity of the immune response to infection, the best antibody immune correlate is likely to be multi-faceted. Numerous antibody specificities, forms, and functions act in concert, as well as temporally, to effectively clear a pathogen and then return the immune system to homeostasis. One path forward to develop a quantitative CoP inclusive of antibody features that represent anti-parasitic functions involves a better understanding of the biophysical properties of antibodies that correspond with protection status.

We hypothesized that antibody subclass and avidity measurements combined with specificity would associate with protection status, similar to previously identified antibody correlates of risk in HIV-1 vaccine efficacy trials^34–37^ and protection status in a Salmonella Typhi vaccine^38^ that included multiple epitope specificities, isotypes, and subclasses. Additionally, we utilized methods with defined precision, specificity, limit of detection and quantitation, and reproducibility^34,39,40^ so that the candidate findings from the current studies could be compared to future larger trials designed to define the protective attributes of antibodies. In our study, the strongest univariate candidate correlate of protection in the standard dose RTS,S/AS01 regimen was NANP6 IgG1 magnitude as measured by BAMA. NANP6 IgG1 was the most predictive of protection status both in univariate logistic regression and in a LASSO penalized regression model. This finding is further supported by a parallel analysis conducted by Young et al., where a predictive modeling framework was used to identify biomarkers of protection from the antibody data generated here combined with cellular, Fc effector function, and transcriptomic immune data from the MAL-068 and MAL-071 studies^41^. In that study, NANP6-targeted antibody dependent cellular phagocytosis and NANP6 IgG1 magnitude were consistently identified as predictive immune response measurements.

We also identified that univariate measurements of IgG1 CSP and IgG3 NANP6 magnitude associated with protection status across the two vaccine studies combined. A prior study found that antibody responses to the HepB component of the vaccine, along with IgG1 and IgG3 to CSP C-terminus and NANP were associated with protection status^42^. In our study, the top candidate correlates associated with protection did not include antibodies to HepB or CSP C-terminus. A higher ratio of cytophilic (IgG1 + IgG3) to noncytophilic (IgG2 + IgG4) was associated with protection^42^. Additionally, it was reported that the association of IgG subclasses with vaccine mediated protection is antigen and subclass dependent, such that IgG3 contributes to protection and IgG2 to malaria risk^43^. IgG4 was previously associated with vaccine efficacy^26^. In our study IgG4 binding to CSP was included in some of the multivariate models; however, this measurement did not reach statistical significance. Across vaccine studies, BLI measurements of total serum CSP, NANP6, NPNA3 and N-interface dissociation phase AUC (AUC_diss_) were associated with protection status. These results are consistent with prior studies that reported that IgG magnitude and avidity against CSP associated with protection status^20,44–46^. However, a quantitative threshold across different populations and vaccine regimens still needs to be defined.

An important characteristic of the generation of protective antibodies by the immune system is the affinity maturation of antibodies driven by exposure to antigen and selection of activated memory B cells in germinal centers. Repeated immunization or exposure to antigen by infection will drive overall affinity maturation to a specific antigen unless an affinity ceiling^47^ is reached or there is an abundance of low affinity naïve B cell precursors that are stimulated. A recent study that examined memory B cell responses in a controlled human malaria infection trial revealed that the efficiency of affinity maturation declined likely due to antigen complexity and the precursor frequency of antigen reactive B cells^48^. Pallikuth et al. demonstrated that early induction of IL-21 secreting CSP specific T follicular helper and CSP specific memory B cell responses likely contributed to the protection in the delayed fractional dose RTS,S AS01 vaccine regimen^49^. Since the CSP protein contains multiple repeating sequences, low affinity antibodies binding to multiple repeats can form a multivalent complex and thus enhance the avidity^50^, which is consistent with the higher NPNA3 responses (Table 3) observed in the protected vaccinees. Protective antibodies induced by other candidate vaccines target the junctional peptide located between the central repeat region and the N terminus, in addition to NANP repeats^7,8^. Notably, we reported earlier that the RTS,S/AS01 vaccine induced antibodies that also cross-react with the junctional peptide (N-interface peptide)^31,32^. In the combined analysis reported here, the N-interface peptide binding AUC_diss_ had a 1.4 to 3.3 fold difference in medians between protected and infected vaccinees and was significantly associated with protection in these two studies. The resulting antibody specificities and avidities associated with protection status were vaccine regimen dependent. For example, we found that the delayed fractional dose arm of RTS,S/AS01 elicited higher avidity CSP and PF16 antibodies associated with protection^32^, highlighting the need to further understand how vaccine dose and timing between immunizations influences the germinal center response and antibody affinity maturation.

Through the antibody Fc domain, antibodies can bind complement and cellular Fc receptors (FcR) to mediate pathogen clearance. The engagement of cell surface FcR by antibodies triggers multiple downstream effector functions, including phagocytosis, antibody dependent cell-mediated cytotoxicity, respiratory burst, and formation of neutrophil extracellular traps (NETs) from neutrophils. A recent analysis detailed that antibody mediated phagocytosis and antibody FcγR3A engagement could predict protection^27^. IgG subclasses have different affinities for cellular Fc receptors and mediate anti-parasitic function through engagement of phagocytes and Natural Killer (NK) cells. The different qualities of the antibody responses reported here including specificity, and IgG1 and IgG3 subclass play key roles in targeting functional antibody responses. It may be possible to tune avidity by different vaccination schedules and adjuvants and enhance the quality and quantity of antibody Fc effector functions. Thus, antibody magnitude, subclass, and avidity measurements together with reported measures of antibody Fc effector functions of phagocytosis and binding to FcγR3A that correlated with protection status are all key immune measurements to test in further immune correlates analyses^27,41^.

In MAL-071, IL-21 secreting CSP specific peripheral T follicular helper (pTfh) cells and memory B cells were associated with protection^49^, indicating that T cell and B cell immune measurements could also substantially contribute to the identification of immune correlates of protection. Kazmin et al. explored a systems biology analysis of the RTS, S/AS01 vaccinees serum and reported that the CSP-specific antibody titers associated with protection status along with enhanced expression of genes associated with B-cells, plasma cells, cell cycle, and a negative association of NK cells modules^51^. Moncunill et al. reported transcriptomic signatures at both baseline and post-vaccination that corresponded to protection status including monocyte-related signatures along with interferon, NF-kB, and Toll-like receptor (TLR)^52^. Du et al.^53^ reported that a transcript ratio of MX2/GPR183 complements CSP antibody titer for distinguishing protected from infected individuals and similar to Moncunill et al. reported a role for interferon signaling. Further studies are needed to determine if the reported gene signatures are linked to the quality and magnitude of the antibody response with different vaccine regimens.

The host genetic background, including FcR polymorphisms and different antibody allotypes, can also alter the biophysical properties of antibody Fc interactions and influence the functional potential of antibodies for parasitic clearance. One study reported an association of HLA alleles with either protection or lack of protection^54^. All of these additional immunological and host genetic factors may explain the vaccine elicited immune heterogeneity we observed across the studies. Understanding individual variation in vaccine responsiveness may be the key to achieving high levels of vaccine efficacy in different populations.

The limitations in our analyses were primarily due to the sample sizes available for each study that limited the power of this study to identify multiple candidate immune correlates. Our findings will need to be validated in subsequent malaria CHMI studies such as MAL-092 (NCT03162614) and in larger field trials assessing baseline immunologic markers and to confirm whether the same immunologic markers correlated with protection in the context of malaria-endemic settings with pre-existing malaria exposure. One such Phase II field study, MAL-094, is currently underway in Ghana and Kenya to assess the efficacy, safety, and immunogenicity of the RTS,S/AS01E vaccine in both the standard dose (RRR) and delayed fractional dose (RR_r) regimens in children aged 5-17 months (NCT03276962). These studies will help to determine the influence of the vaccine interval and dose on modulating specific immunity and thus efficacy. It is also possible that the antibody measurements identified here are not directly responsible for mediating protection and may be a surrogate for another immune parameter that is a mechanistic CoP.

In conclusion, we evaluated antibody specificity, subclass and avidity as measures of the quality of the vaccine elicited immune response to RTS,S/AS01 vaccination and as correlates of protection status. We uniquely identified antibody biophysical measurements of specificity, subclass and avidity that correlate with protection status and contribute to an enhanced understanding of the potential mechanisms underpinning the protection provided by the RTS,S vaccine. These data provide a set of immunological markers that together can be confirmed in field trials as a CoP for RTS,S or other malaria vaccines and inform the next generation of effective and durable malaria vaccines.

## Materials and methods

### Antigens and monoclonal antibodies

Full length recombinant CSP (CSP) containing the N-terminal region, 3 NVDP and 19 NANP repeats followed by the C-terminal region was obtained from Dr. Sheetij Dutta, Walter Reed Army Institute of Research (WRAIR)^55^. Synthetic peptides NANP6 and PF16 corresponding to the central repeat and carboxy terminal regions of CSP respectively were made with an amino terminal biotin-Aminohexanoic acid (biotin-Ahx) tag. NANP6 (biotin-Ahx-NANPNANPNANPNANPNANPNANP) and the negative control peptide antigen C1 (Biotin-KKMQEDVISL WDQSLKPCVK LTPLCV) were obtained from CPC Scientific (Sunnyvale, CA) for the BLI measurements. PF16 (biotin-Ahx- EPSDKHIKEY LNKIQNSLST EWSPCSVTCG NGIQVRIKPG SANKPKDELD YANDIEKKIC KMEKCS with an amidated carboxy terminal) was procured from Biomatik (Cambridge, ON, Canada). NPNA3 (biotin-Ahx-NPNANPNANPNA with an amidated carboxy terminal) and N-interface (biotin-Ahx-KQPADGNPDPNANPN with an amidated carboxy terminal) were custom made by CPC Scientific. The NANP6 (EP070034; NANPNANPNANPNANPNANPNANPC) peptide used in BAMA assay was a product of Biomatik (Cambridge, ON, Canada). Vaccine matched Hepatitis B (HepB) antigen was obtained from GlaxoSmithKline. The negative control used in BLI assays, Ovalbumin-biotin was purchased from Galab Laboratories (Hamburg, Germany). Recombinant monoclonal antibodies (mAbs) AB334 and AB236 that are specific for the central repeat and C-terminal regions of CSP, respectively^39^, were used as standards for quality control tracking of BAMA and BLI avidity assays performed over several days. For BAMA assays, AB334 and AB236 were titrated 3-fold, 11 places, starting at 10 µg/ml and 30 µg/ml respectively. For BLI assays, AB334 at 1, 3.75, 7.5, 15 and 50 µg/ml concentrations and AB236 at 1, 2, 3.75, 7.5 and 50 µg/ml concentrations were used to construct standard curves.

### Study samples

Samples from participants in the MAL-068 (NCT01366534) and MAL-071 (NCT01857869) clinical trials were collected following informed consent. The efficacy and immunological evaluations for both clinical trials were reported previously^16,20,26,30^. The study protocols were approved by the WRAIR Institutional Review Board and PATH-Malaria Vaccine Initiative’s Western IRB and all participants provided informed consent. Retrospective analysis presented in this study was performed with approval from the Duke Medicine Institutional Review Board for Clinical Investigations (Protocol Pro00074497). All study participants had previously provided consent for future use of samples for research, and all samples were de-identified.

### Binding antibody multiplex assay (BAMA)

We evaluated antibody binding to full-length CSP, NANP6 (EP070034), NPNA3, PF16, and HepB using a custom BAMA^40,56,57^. Vaccinee sera were diluted in BAMA assay diluent (1% milk-blotto, 5% normal goat serum, 0.05% Tween-20) and incubated with antigen-coupled microspheres for 30 min. Samples were then incubated with either anti-human IgG1 (BioLegend, clone 12G8G11, Catalog number: 409904), anti-human IgG2 (Southern Biotech, clone HP6002, Catalog number:9070-01), anti-human IgG3 (Invitrogen, clone HP6047, Catalog number:053600), or anti-human IgG4 (BD Pharmingen, clone JDC-14, Catalog number:555878) at a final concentration of 4 µg/mL in assay diluent, followed by Goat Anti-Mouse IgG, Human ads-PE (Southern Biotech, clone: HP6002, Catalog number:1030-09) at a final concentration of 4 µg/mL in assay diluent and detected on a Bioplex 200 (Bio-Rad). Controls for assays included a titrated purified human subclass specific standard curves or antigen-specific monoclonals and purified subclass-specific coupled beads. Negative controls in each assay included normal human reference serum (Sigma-Aldrich) and blank (no-antigen) beads. Each experiment was performed using Good Clinical Laboratory Practice–compliant conditions, including tracking of positive controls by Levey-Jennings charts. Positive responders were defined as samples with a mean fluorescence intensity (MFI) > 100, MFI*Dilution Factor > 95th percentile of all baselines within study, and MFI*Dilution Factor > 3x baseline. *Antibody avidity*: Assessment of antibody AI was determined by BAMA with the following modifications: After formation of antigen/antibody immune complexes, a 15-min dissociation step (Na-Citrate, pH 4.0, Teknova; CIT)^58^ at room temperature (20–23 °C) was included prior to addition of secondary detection antibody. Retained binding magnitude was expressed as AI (AI = MFI (CIT)/MFI (PBS)*100) and used as a measurement of antibody avidity in the statistical models. AI was calculated only in cases where binding response was positive according to pre-set criteria above. For multivariate analyses, AI was set to 0 for negative responses. AI was reported for samples in the linear range where AI confirmed within 10% across assays and/or sample dilution factors. Samples that did not meet the pre-set criteria were reported as indeterminate for AI measurements. IgG2 PF16 and HepB AI and IgG4 NANP6, NPNA3, PF16, and HepB AI were not included in analyses. Positive response rates by regimen and protection status for the corresponding binding magnitude measurements were all less than 30%, leading to few participants with quantifiable AI.

### Biolayer interferometry (BLI) avidity assay

The BLI assay for monitoring the avidity of malaria vaccine induced antibodies^39^ was used to measure the RTS,S/AS01 vaccine induced serum antibody binding responses and the off rates of interaction with CSP, NANP6, NPNA3, PF16, and N-interface. BLI assays were carried out using Fortebio OctetRed 384 instruments and biosensors (Fortebio- Biologics by Molecular Devices, San Jose, CA). Both data acquisition and analyses were performed with United States Food and Drug Administration’s Title 21 Code of Federal Regulations Part 11 (FDA Title 21 CFR Part 11) compliant software versions (Data Acquisition 9.0 and Data Analysis 9.0/10.0 packages). Vaccinee sera from both studies were tested for antigen binding at 1:50 dilution in phosphate buffered saline (PBS) pH 7.4 (Gibco, Thermo Fisher Scientific, Waltham, MA) in triplicate. Antigens NANP6, PF16 and negative control peptide C1 were loaded onto streptavidin biosensors (threshold level set to not exceed Δλ = 1 nm) whe.reas CSP and negative control ovalbumin were coupled to the amine reactive (AR2G) biosensors (threshold level set to not exceed Δλ = 0.7 nm). Antigens NPNA3, N-interface and negative control peptide C1 were loaded onto streptavidin sensors with the threshold level set to not exceed Δλ = 0.1 nm and the vaccinee sera were diluted into 1x kinetics buffer (Fortebio-Biologics by Molecular Devices, San Jose, CA). The 1:50 diluted vaccinee sera binding to the parallel reference sensors immobilized with negative control antigens were subtracted to obtain antigen specific binding time courses. Binding responses (Δλ averaged at the last 5 s of association phase) and the off rates of vaccinee sera binding were determined. Antigen specific positivity limit (mean plus three times standard deviation of reference human serum binding response) and lower limit of quantitation (LLOQ; empirically determined antigen specific binding response above which off rate can be measured reliably for standard antibody) were applied in quality controlling of data. This involved ensuring that the percent coefficient of variation (%CV) in binding responses that are positive for a given antigen was <20 and the variation in off rates were ≤2 fold for sera with responses greater than LLOQ. For correlation analyses and the summary values in all tables, positive responders with binding responses below LLOQ were assigned an off rate of 1 × 10^-2^ s^-1^. The AUC of the dissociation curve (AUC_diss_) was calculated using the specific binding time course data with the R package ‘caTools’ to get the trapezoidal rule estimate of the area under the response magnitude curve over time.

### Statistical analyses

Statistical analyses were performed using R statistical software (version 4.0.4; R Foundation for Statistical Computing, Vienna, Austria). In order to compare immune responses between protected and infected vaccinees, binomial logistic regression models were fit to each variable independently on combined data from both studies, with a term in the model to adjust for regimen (Protection~Regimen + Measurement). Regression was performed using the R function ‘glm’. Prior to analysis, each immune measurement was log-transformed and scaled to have mean 0 and standard deviation 1. The Benjamini–Hochberg procedure was used to control the false discovery rate (FDR), and effects were considered to be statistically significant if FDR-adjusted *p*-value < 0.2 and *p*-value < 0.05 before FDR-adjustment. Variance inflation factors for the full logistic regression model including all 22 measurements which were measured in both studies were calculated using the R package ‘car’.

Comparisons of immune responses between MAL-068 RRR and ARR vaccinees, as shown in Figs. S1–S4, were performed using the Mann-Whitney U test. *P*-values are two-sided and effects were considered to be statistically significant for *p*-values < 0.05. Statistical comparisons of groups are shown only for day 77, the day of challenge, and *p* > 0.05 for comparisons where a *p*-value is not mentioned. No adjustments were made for multiple testing due to the small sample sizes and exploratory nature of these comparisons.

For all boxplots contained in main and supplementary figures, the lower and upper hinges of the box correspond to the 25th and 75th percentiles, with a line at the median. The lower and upper whisker extends from the box hinges to the smallest and largest values, respectively, which are within 1.5 * IQR of the hinge (where IQR, the inter-quartile range, is equal to the distance between the 25th and 75th percentiles).

### Data Imputation

Prior to multivariate analyses, missing values were imputed using the *k*-nearest neighbors method in the R package ‘caret’. No variable had > 15% missing data, with only four variables having ≥ 5% missing data (IgG1 CSP AI, IgG2 CSP AI, IgG1 NANP6 AI, and IgG2 NANP6 AI).

### Least absolute shrinkage and selection operator (LASSO)

Using the 21 measurements which were measured in both studies (after excluding CSP AUC_diss_, which had VIF > 10), binary logistic regression with LASSO regularization was fit to the combined data from both studies using the R package ‘glmnet’^59^. RR_r and ARR indicators (0 or 1 value, where 0 = no and 1 = yes) were included to adjust for regimen. RRR is not explicitly included in the regression, but RRR vaccination is assumed if both RR_r and ARR indicators are equal to 0. 1000 replicates of nested 5-fold cross-validation was performed such that λ was selected within the inner loop (either lambda.min, the value which minimized leave-one-out cross-validation misclassification or lambda.1se, the largest value of λ such that misclassification error was within 1 standard error of the minimum) while model performance was assessed in the outer loop. For final regression on the entire data set, lambda.min and lambda.1se values were obtained using leave-one-out cross-validation, with models shown for the entire path of λ values.

As a sensitivity analysis to ensure that data imputation was not influencing analysis results, the same procedure was applied to the subset of 78 (of 92) subjects with no missing data (IgG1 CSP AI and IgG2 CSP AI removed due to a higher percentage of missing values). The 14 subjects with at least one missing immune measurement were well balanced with respect to study, regimen arm, and protection status. The ranking of features, cross-validation accuracy, and models corresponding to lambda.min and lambda.1se were similar to those using the imputed data.

### Reporting summary

Further information on research design is available in the Nature Research Reporting Summary linked to this article.

## Supplementary information

Supplementary Information

Reporting Summary

## Data Availability

All data generated or analyzed during this study are included in this published article (and its supplementary information files) or in other publications described. All raw data are available upon request.
