# Supplementary material for: Subclass and avidity of circumsporozoite protein specific antibodies associate with protection status against malaria infection
Source: NPJ Vaccines. 2021 Aug 30;6:110. doi: 10.1038/s41541-021-00372-x (PMC8405700; doi:10.1038/s41541-021-00372-x)
Supplement: Supplementary file 1 — Supplementary Information [file 41541_2021_372_MOESM1_ESM.pdf]

Supplementary Information

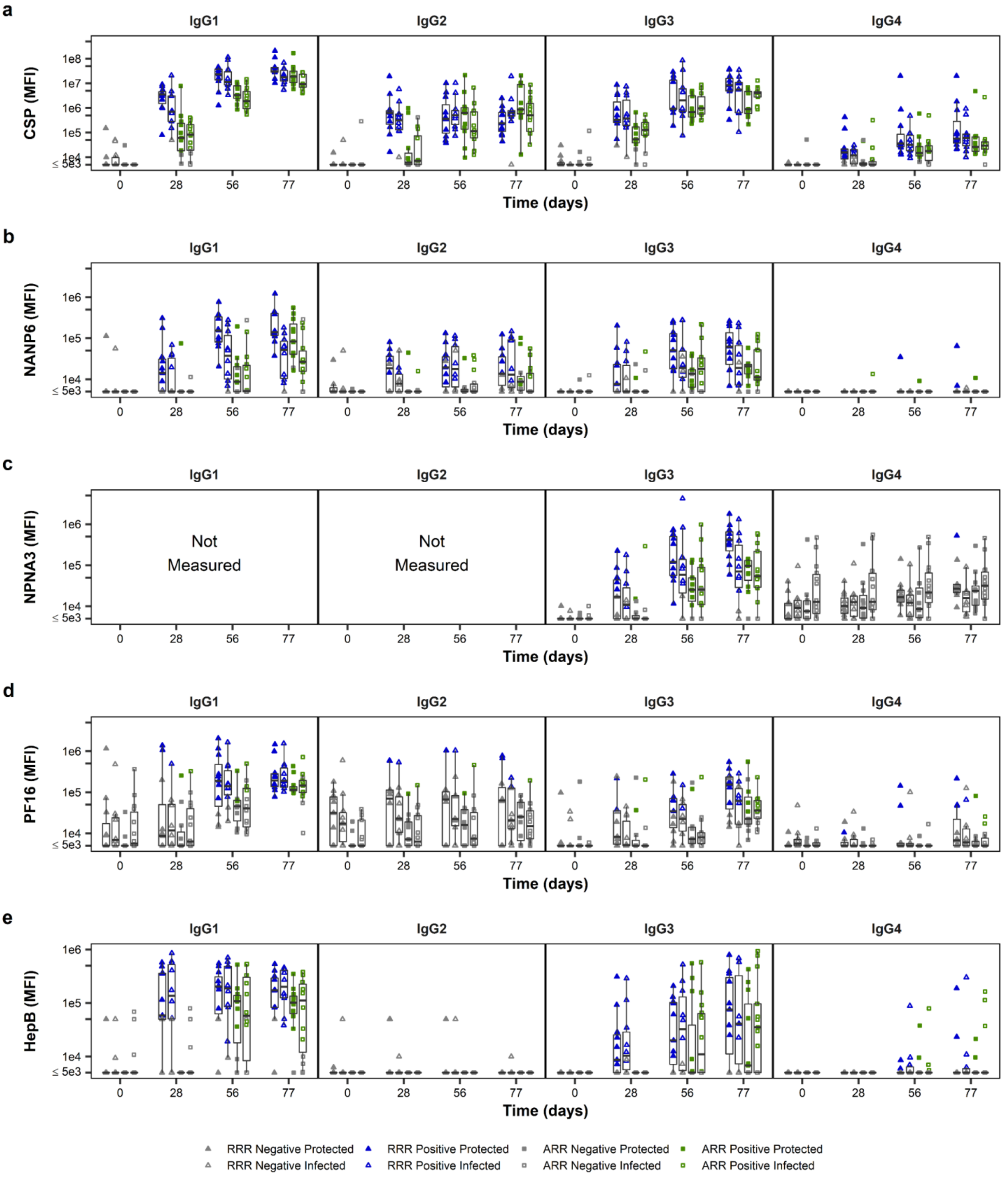

Supplementary Figure 1: ARR and RRR regimens elicit robust CSP specific IgG responses in MAL-068 study. The IgG1, IgG2, IgG3, and IgG4 subclass antibodies binding magnitude to CSP (a), NANP6 (b), NPNA3 (c), PF16 (d), and HepB (e) are presented as MFI * Dilution Factor values at days 0 (baseline), 28 (1 month post 1st vaccination), 56 (1 month post 2nd vaccination), and 77 (1 month post 3rd vaccination/day of challenge) for vaccine responders in the ARR (green squares) and RRR (blue triangles) regimens, shown by protection status. At day 77, RRR vaccinees had higher IgG1, IgG3, and IgG4 binding magnitude to CSP (a; $p = 0.009$, $p = 0.026$ and $p = 0.018$, respectively), higher IgG2 and

IgG4 binding magnitude to NANP6 (b; p = 0.029 and p = 0.027, respectively), higher IgG3 binding magnitude to NPNA3 (c, p = 0.020), higher IgG1 and IgG4 binding to PF16 (d; p = 0.016 and p = 0.032, respectively), and higher IgG1 binding magnitude to HepB (e, p = 0.032). Non-responders are represented by grey squares (ARR) or grey triangles (RRR), with positive vaccine-induced response calls determined as described in the Methods section. The lower and upper hinges of the boxplots correspond to the 25th and 75th percentiles, with a line at the median. The lower and upper whisker extends from the box hinges to the smallest and largest values, respectively, which are within 1.5 * IQR of the hinge (where IQR, the inter-quartile range, is equal to the distance between the 25th and 75th percentiles).

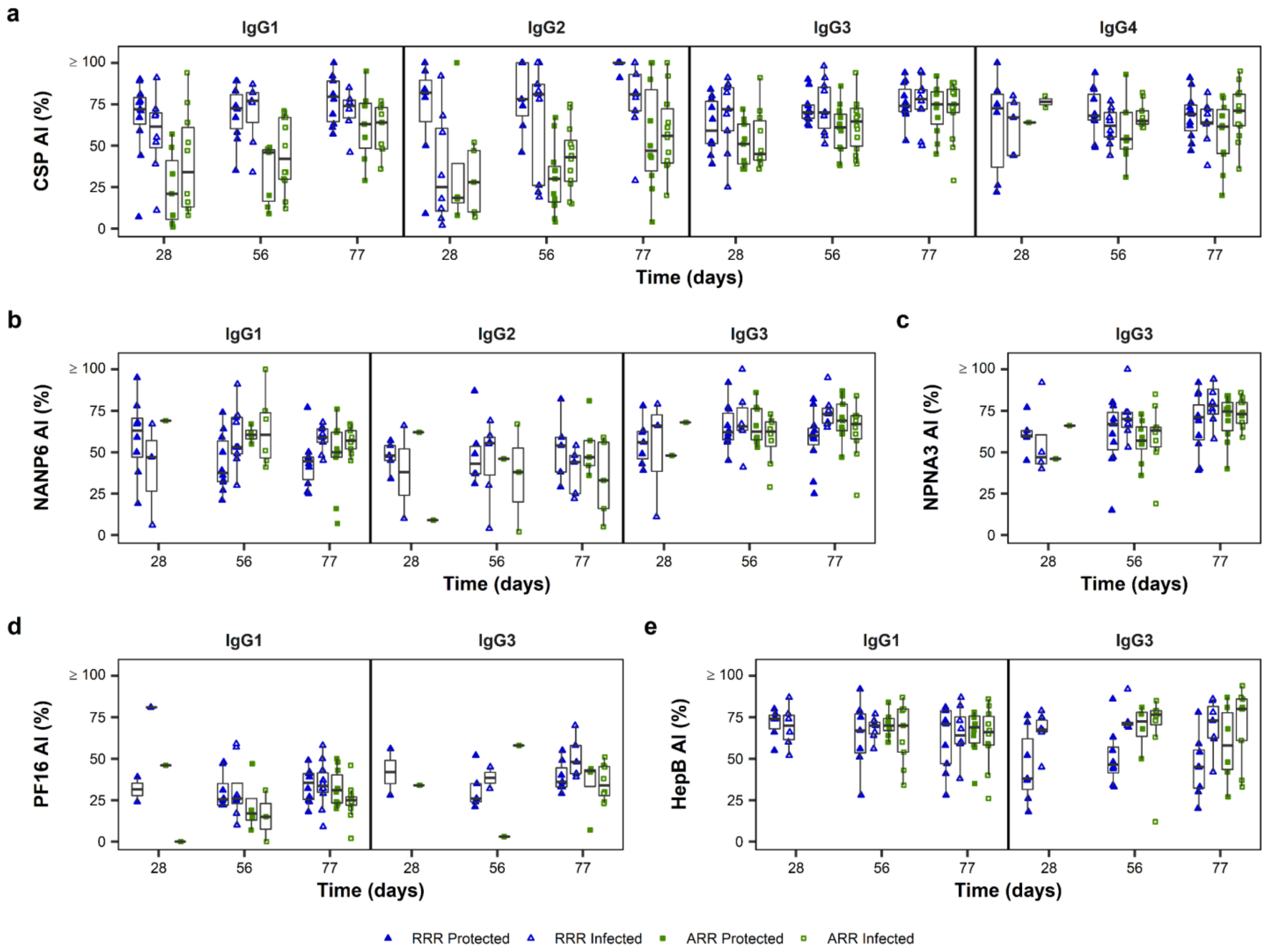

Supplementary Figure 2: RRR regimen elicits antibodies with higher avidity index compared with the ARR regimen in MAL-068 study. The IgG1, IgG2, IgG3, and IgG4 subclass antibodies AI for positive vaccine responders to CSP (a), NANP6 (b), NPNA3 (c), PF16 (d), and HepB (e) is presented for ARR (green squares) and RRR (blue triangles) regimens by protection status. At day 77, RRR vaccinees had higher IgG1 and IgG2 CSP AI compared to ARR vaccinees (a; $p = 0.027$ and $p = 0.001$, respectively). For IgG2 PF16 and HepB and IgG4 NANP6, NPNA3, PF16, and HepB, positive response rates were too low to determine AI values (see methods for more details). The lower and upper hinges of the boxplots correspond to the 25th and 75th percentiles, with a line at the median. The lower and upper whisker extends from the box hinges to the smallest and largest values, respectively, which are within 1.5 * IQR of the hinge (where IQR, the inter-quartile range, is equal to the distance between the 25th and 75th percentiles).

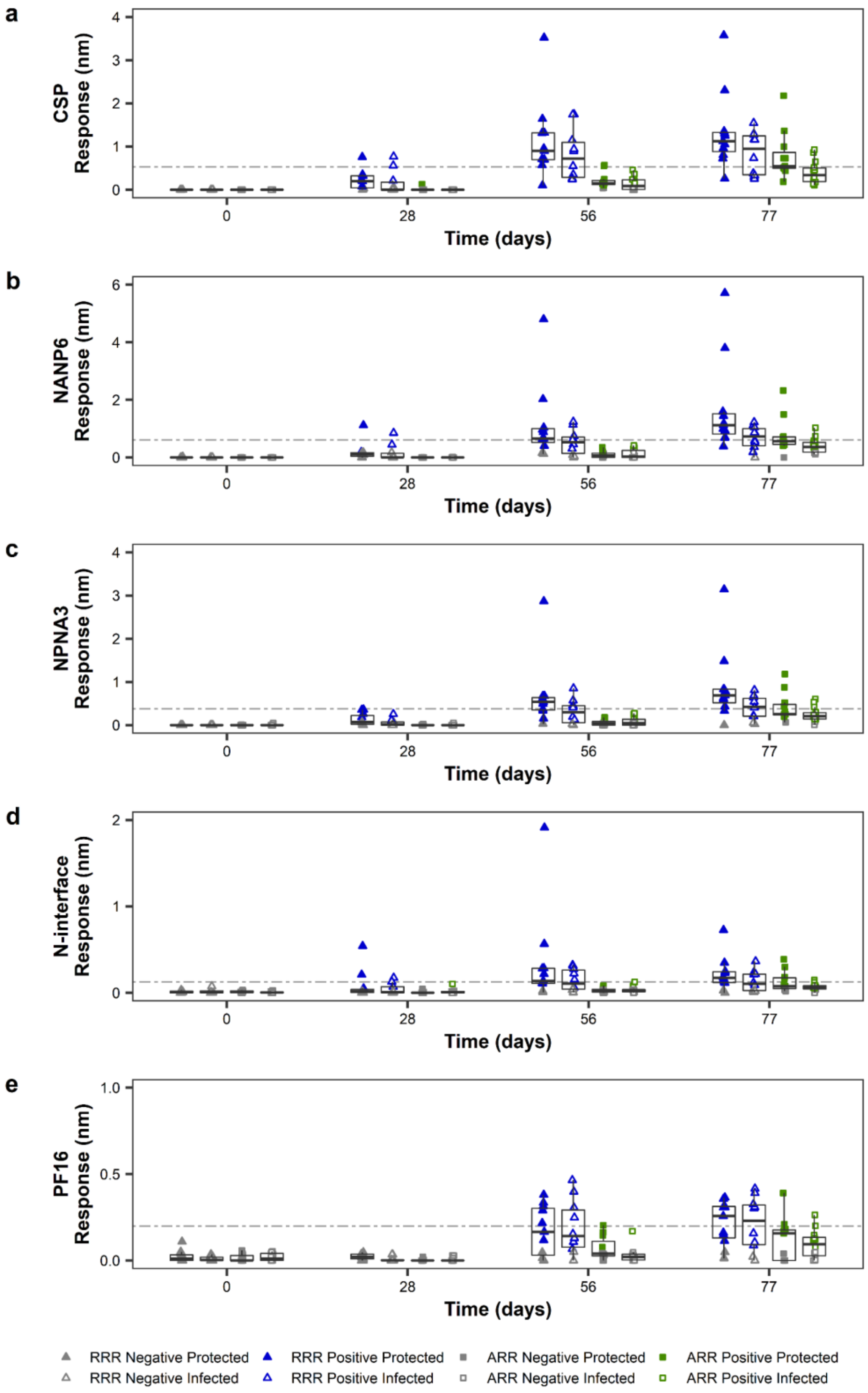

Supplementary Figure 3: Higher magnitude antibody responses are induced by RTS,S/AS01 regimen. Specific binding responses of vaccinees' sera are shown for ARR (green squares) and RRR groups (blue triangles) at days 0 (baseline), 28 (1 month post 1st vaccination), 56 (1 month post 2nd vaccination), and 77 (1 month post 3rd vaccination/day of challenge). At day 77, RRR vaccinees had significantly higher binding responses to CSP (a, $p = 0.003$), CSP central repeat region peptides NANP6 (b, $p = 0.003$) and NPNA3 (c, $p = 0.014$), N-interface (d, $p = 0.070$) and the CSP C-terminal region peptide PF16 (e, $p = 0.007$) antigens. Grey colored symbols indicate negative responders, with positive vaccine-induced response calls determined as described in the Methods section. Dashed grey lines in each panel denote the response levels corresponding to LLOQ. The lower and upper hinges of the boxplots correspond to the 25th and 75th percentiles, with a line at the median. The lower and upper whisker extends from the box hinges to the smallest and

largest values, respectively, which are within 1.5 * IQR of the hinge (where IQR, the inter-quartile range, is equal to the distance between the 25th and 75th percentiles).

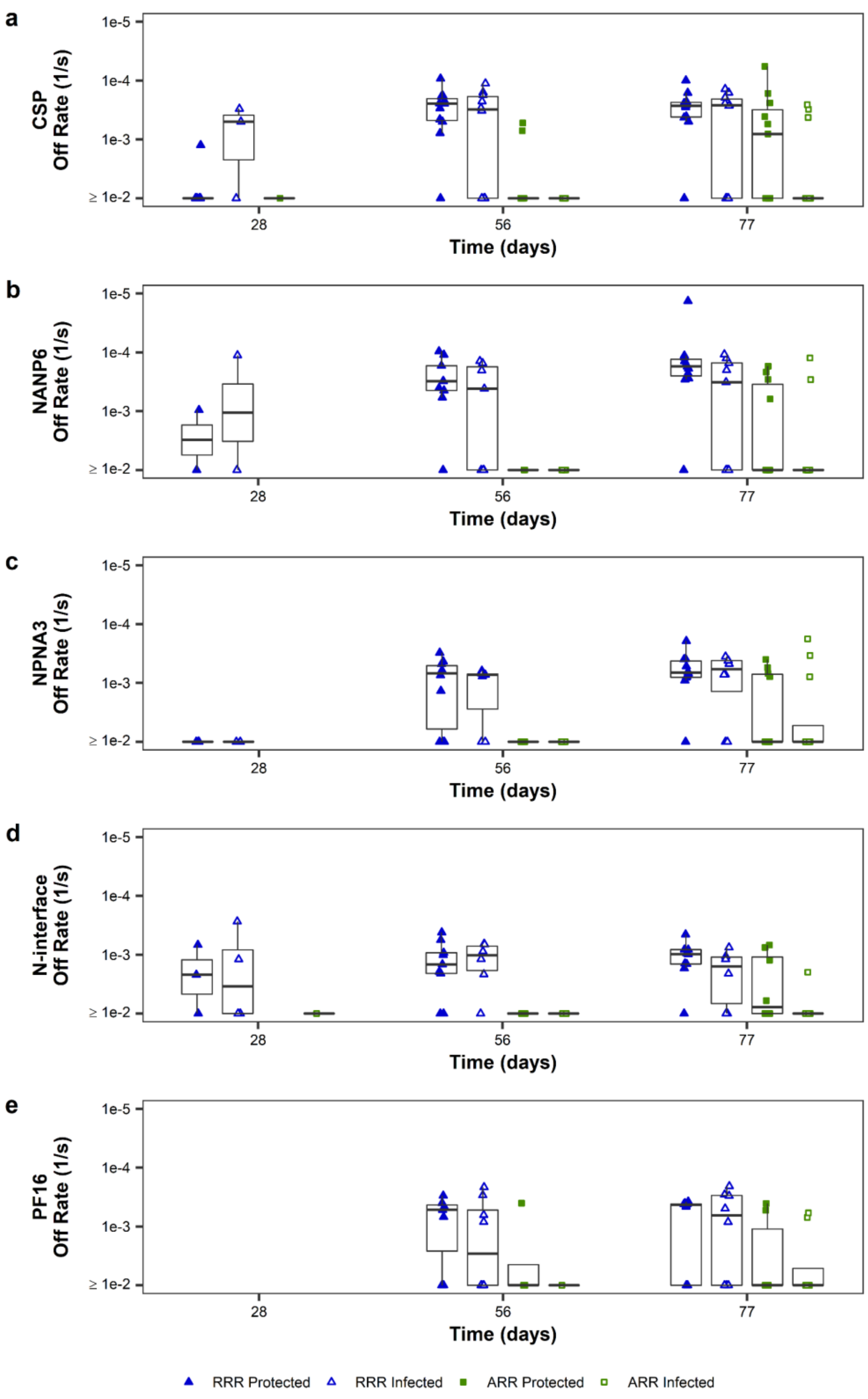

Supplementary Figure 4: RTS,S/AS01 regimen induces higher avidity antibodies. The off rates of vaccinees sera at different time points binding to CSP (a), CSP central repeat region peptides NANP6 (b) and NPNA3 (c), N-interface (d) and the CSP C-terminal region peptide PF16 (e) antigens are shown for ARR group (green squares) and RRR group (blue triangles). Off rates were significantly slower in the RRR vaccinees compared to the ARR vaccinees for binding to CSP (a, $p = 0.004$), NANP6 (b, $p = 0.002$), NPNA3 (c, $p = 0.006$), N-interface (d, $p = 0.007$) and PF16 (e, $p = 0.018$). Off rates were set to 1×10^{-2} s^{-1} if the binding responses of sera (shown in **Figure S3**) were positive but below LLOQ for a given antigen. The lower and upper hinges of the boxplots correspond to the 25th and 75th percentiles, with a line at the median. The lower and upper whisker extends from the box hinges to the smallest and largest values, respectively, which are within 1.5 * IQR of the hinge (where IQR, the inter-quartile range, is equal to the distance between the 25th and 75th percentiles).

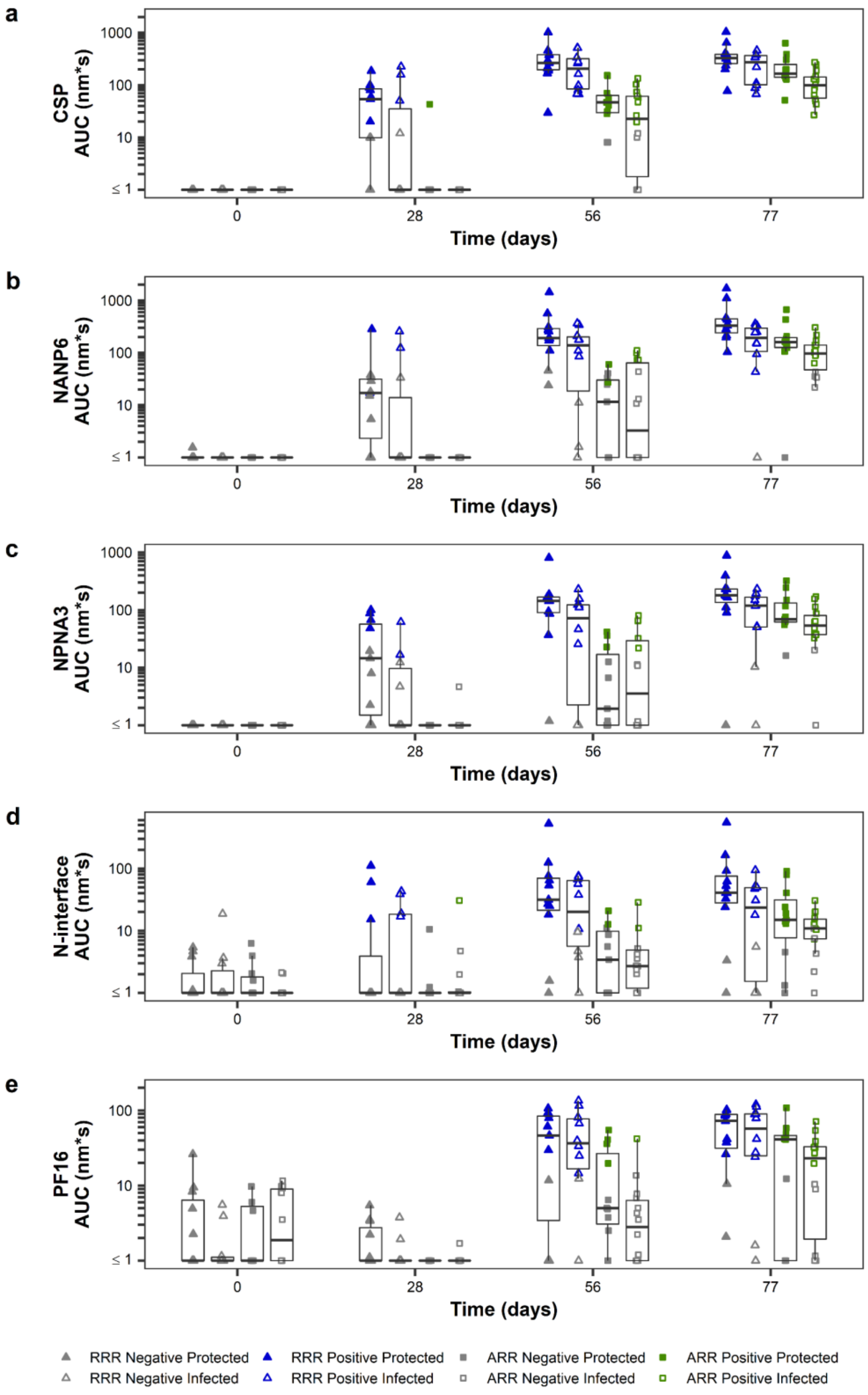

Supplementary Figure 5: Comparison of overall dynamics of antigen binding of serum antibodies induced by ARR and RRR regimens. The AUC_{diss} of vaccinees sera drawn at different time points are shown for binding to CSP (a), CSP central repeat region peptides NANP6 (b) and NPNA3 (c), N-interface (d) and the CSP C-terminal region peptide PF16 (e) antigens are shown for ARR group (green squares) and RRR group (blue triangles) respectively. At day 77, RRR vaccinees had significantly AUC_{diss} for CSP (a, $p = 0.002$), CSP central repeat region peptides NANP6 (b, $p = 0.003$) and NPNA3 (c, $p = 0.018$), N-interface (d, $p = 0.042$) and the CSP C-terminal region peptide PF16 (e, $p = 0.007$) antigens. Grey colored symbols indicate negative responders. The lower and upper hinges of the boxplots correspond to the 25th and 75th percentiles, with a line at the median. The lower and upper whisker extends from the box hinges to the smallest and largest values, respectively, which are within 1.5 * IQR of the hinge (where IQR, the inter-quartile range, is equal to the distance between the 25th and 75th percentiles).

Supplementary Table 1: Intra-assay Spearman rank correlation coefficients for BAMA measurements.

	IgG1 CSP	IgG1 CSP AI	IgG1 NANP6	IgG1 NANP6 AI	IgG2 CSP	IgG2 CSP AI	IgG2 NANP6	IgG2 NANP6 AI	IgG3 CSP	IgG3 CSP AI	IgG3 NANP6	IgG3 NANP6 AI	IgG3 HepB	IgG3 PF16	IgG4 CSP	IgG4 CSP AI	IgG4 NANP6
IgG1 CSP	1.00	0.17	0.54	0.15	-0.08	0.31	0.06	0.02	0.24	0.23	0.44	0.20	0.20	0.15	0.06	0.10	0.00
IgG1 CSP AI	0.17	1.00	0.46	0.34	-0.09	0.33	0.10	0.24	-0.06	0.50	0.15	0.09	0.27	0.03	0.43	0.31	0.21
IgG1 NANP6	0.54	0.46	1.00	0.25	-0.06	0.40	0.17	0.27	0.01	0.46	0.37	0.20	0.20	0.08	0.30	0.23	0.12
IgG1 NANP6 AI	0.15	0.34	0.25	1.00	0.16	0.21	0.03	0.27	-0.19	0.40	0.09	0.22	0.23	-0.12	0.27	0.18	-0.30
IgG2 CSP	-0.08	-0.09	-0.06	0.16	1.00	-0.04	0.47	0.41	-0.02	-0.11	0.07	0.07	-0.01	-0.08	0.08	-0.13	-0.10
IgG2 CSP AI	0.31	0.33	0.40	0.21	-0.04	1.00	0.30	0.35	0.21	0.17	0.36	0.16	0.28	0.14	0.32	0.19	0.17
IgG2 NANP6	0.06	0.10	0.17	0.03	0.47	0.30	1.00	0.71	-0.11	0.12	0.15	0.08	0.03	-0.14	0.40	-0.05	0.13
IgG2 NANP6 AI	0.02	0.24	0.27	0.27	0.41	0.35	0.71	1.00	-0.20	0.20	0.19	0.11	0.10	-0.24	0.46	0.02	0.00
IgG3 CSP	0.24	-0.06	0.01	-0.19	-0.02	0.21	-0.11	-0.20	1.00	-0.16	0.63	0.27	0.31	0.66	-0.06	-0.07	0.26
IgG3 CSP AI	0.23	0.50	0.46	0.40	-0.11	0.17	0.12	0.20	-0.16	1.00	0.26	0.29	0.06	0.03	0.26	0.32	-0.12
IgG3 NANP6	0.44	0.15	0.37	0.09	0.07	0.36	0.15	0.19	0.63	0.26	1.00	0.54	0.29	0.34	0.10	0.18	0.16
IgG3 NANP6 AI	0.20	0.09	0.20	0.22	0.07	0.16	0.08	0.11	0.27	0.29	0.54	1.00	0.27	0.17	0.07	0.23	-0.01
IgG3 HepB	0.20	0.27	0.20	0.23	-0.01	0.28	0.03	0.10	0.31	0.06	0.29	0.27	1.00	0.31	0.32	0.13	0.18
IgG3 PF16	0.15	0.03	0.08	-0.12	-0.08	0.14	-0.14	-0.24	0.66	0.03	0.34	0.17	0.31	1.00	0.04	-0.08	0.25
IgG4 CSP	0.06	0.43	0.30	0.27	0.08	0.32	0.40	0.46	-0.06	0.26	0.10	0.07	0.32	0.04	1.00	-0.10	0.37
IgG4 CSP AI	0.10	0.31	0.23	0.18	-0.13	0.19	-0.05	0.02	-0.07	0.32	0.18	0.23	0.13	-0.08	-0.10	1.00	0.06
IgG4 NANP6	0.00	0.21	0.12	-0.30	-0.10	0.17	0.13	0.00	0.26	-0.12	0.16	-0.01	0.18	0.25	0.37	0.06	1.00

Supplementary Table 2: Intra-assay Spearman rank correlation coefficients for BLI measurements

	CSP (nm)	CSP AUC	CSP Off rate	NANP6 (nm)	NANP6 AUC	NANP6 Off rate	NPNA3 (nm)	NPNA3 AUC	NPNA3 Off rate	N-interface (nm)	N-interface AUC	N-interface Off rate	PF16 (nm)	PF16 AUC	PF16 Off rate
CSP (nm)	1.00	1.00	0.66	0.93	0.94	0.78	0.79	0.78	0.65	0.68	0.65	0.58	0.73	0.72	0.66
CSP AUC	1.00	1.00	0.68	0.93	0.94	0.79	0.79	0.78	0.64	0.69	0.66	0.58	0.74	0.73	0.67
CSP Off rate	0.66	0.68	1.00	0.58	0.59	0.68	0.47	0.48	0.37	0.49	0.48	0.44	0.61	0.62	0.54
NANP6 (nm)	0.93	0.93	0.58	1.00	1.00	0.76	0.78	0.75	0.57	0.71	0.67	0.56	0.59	0.58	0.53
NANP6 AUC	0.94	0.94	0.59	1.00	1.00	0.78	0.79	0.77	0.59	0.72	0.68	0.57	0.61	0.60	0.54
NANP6 Off rate	0.78	0.79	0.68	0.76	0.78	1.00	0.69	0.69	0.58	0.64	0.61	0.56	0.55	0.54	0.50
NPNA3 (nm)	0.79	0.79	0.47	0.78	0.79	0.69	1.00	1.00	0.82	0.68	0.65	0.58	0.50	0.49	0.43
NPNA3 AUC	0.78	0.78	0.48	0.75	0.77	0.69	1.00	1.00	0.83	0.68	0.65	0.58	0.51	0.51	0.45
NPNA3 Off rate	0.65	0.64	0.37	0.57	0.59	0.58	0.82	0.83	1.00	0.52	0.51	0.48	0.41	0.41	0.37
N-interface (nm)	0.68	0.69	0.49	0.71	0.72	0.64	0.68	0.68	0.52	1.00	0.99	0.84	0.59	0.59	0.55
N-interface AUC	0.65	0.66	0.48	0.67	0.68	0.61	0.65	0.65	0.51	0.99	1.00	0.84	0.58	0.58	0.55
N-interface Off rate	0.58	0.58	0.44	0.56	0.57	0.56	0.58	0.58	0.48	0.84	0.84	1.00	0.53	0.54	0.49
PF16 (nm)	0.73	0.74	0.61	0.59	0.61	0.55	0.50	0.51	0.41	0.59	0.58	0.53	1.00	1.00	0.84
PF16 AUC	0.72	0.73	0.62	0.58	0.60	0.54	0.49	0.51	0.41	0.59	0.58	0.54	1.00	1.00	0.85
PF16 Off rate	0.66	0.67	0.54	0.53	0.54	0.50	0.43	0.45	0.37	0.55	0.55	0.49	0.84	0.85	1.00

Supplementary Table 3: Inter-assay Spearman rank correlation coefficients for BAMA and BLI measurements

	CSP (nm)	CSP AUC	CSP Off rate	NANP6 (nm)	NANP6 AUC	NANP6 Off rate	NPNA3 (nm)	NPNA3 AUC	NPNA3 Off rate	N-interface (nm)	N-interface AUC	N-interface Off rate	PF16 (nm)	PF16 AUC	PF16 Off rate
IgG1 CSP	0.84	0.84	0.52	0.74	0.75	0.61	0.65	0.64	0.54	0.58	0.55	0.51	0.75	0.75	0.65
IgG1 CSP AI	0.25	0.28	0.53	0.27	0.28	0.36	0.21	0.23	0.23	0.48	0.49	0.43	0.26	0.26	0.29
IgG1 NANP6	0.64	0.65	0.59	0.67	0.68	0.60	0.56	0.55	0.37	0.47	0.47	0.39	0.38	0.39	0.36
IgG1 NANP6 AI	0.29	0.30	0.45	0.31	0.31	0.22	0.09	0.10	-0.01	0.22	0.20	0.10	0.23	0.23	0.20
IgG2 CSP	0.08	0.07	0.00	0.06	0.06	0.02	0.06	0.05	0.01	0.11	0.11	0.14	-0.05	-0.06	-0.05
IgG2 CSP AI	0.48	0.50	0.40	0.48	0.48	0.42	0.42	0.44	0.31	0.36	0.33	0.24	0.33	0.32	0.29
IgG2 NANP6	0.31	0.31	0.17	0.35	0.34	0.33	0.19	0.17	0.19	0.24	0.23	0.22	0.09	0.07	0.10
IgG2 NANP6 AI	0.26	0.27	0.24	0.34	0.33	0.24	0.06	0.05	0.02	0.19	0.18	0.11	0.04	0.03	0.10
IgG3 CSP	0.13	0.13	0.00	0.12	0.13	0.16	0.23	0.24	0.16	0.22	0.20	0.18	0.17	0.17	0.11
IgG3 CSP AI	0.25	0.26	0.48	0.25	0.26	0.23	0.09	0.08	0.04	0.25	0.25	0.11	0.23	0.24	0.26
IgG3 NANP6	0.40	0.41	0.27	0.44	0.44	0.37	0.39	0.38	0.18	0.36	0.32	0.27	0.24	0.24	0.23
IgG3 NANP6 AI	0.18	0.18	0.31	0.19	0.20	0.17	0.12	0.12	-0.05	0.21	0.17	0.09	0.12	0.13	0.17
IgG3 HepB	0.33	0.33	0.40	0.30	0.30	0.37	0.20	0.22	0.03	0.34	0.35	0.24	0.36	0.35	0.39
IgG3 PF16	0.03	0.03	0.05	-0.02	0.00	0.12	0.09	0.11	0.01	0.19	0.20	0.22	0.18	0.19	0.12
IgG4 CSP	0.29	0.30	0.43	0.25	0.25	0.21	0.13	0.14	0.07	0.16	0.18	0.21	0.29	0.28	0.34
IgG4 CSP AI	0.10	0.12	0.24	0.16	0.16	0.18	0.21	0.23	0.11	0.21	0.18	0.02	0.15	0.16	0.10
IgG4 NANP6	0.05	0.04	0.03	0.00	0.00	0.11	0.17	0.19	0.19	0.09	0.11	0.17	0.09	0.09	0.09
