# Supplementary material for: Subclass and avidity of circumsporozoite protein specific antibodies associate with protection status against malaria infection
Source: NPJ Vaccines. 2021 Aug 30;6:110. doi: 10.1038/s41541-021-00372-x (PMC8405700; doi:10.1038/s41541-021-00372-x)
Supplement: Supplementary file 2 — Reporting Summary [file 41541_2021_372_MOESM2_ESM.pdf]

nature research

Corresponding author(s): Drs. Kelly E. Seaton, Georgia D. Tomaras, S. Moses Dennison

Last updated by author(s): Jul 19, 2021

Reporting Summary

Nature Research wishes to improve the reproducibility of the work that we publish. This form provides structure for consistency and transparency in reporting. For further information on Nature Research policies, see our Editorial Policies and the Editorial Policy Checklist.

Statistics

For all statistical analyses, confirm that the following items are present in the figure legend, table legend, main text, or Methods section.

n/a	Confirmed	
☐	☒	The exact sample size (*n*) for each experimental group/condition, given as a discrete number and unit of measurement
☐	☒	A statement on whether measurements were taken from distinct samples or whether the same sample was measured repeatedly
☐	☒	The statistical test(s) used AND whether they are one- or two-sided<br>*Only common tests should be described solely by name; describe more complex techniques in the Methods section.*
☐	☒	A description of all covariates tested
☐	☒	A description of any assumptions or corrections, such as tests of normality and adjustment for multiple comparisons
☐	☒	A full description of the statistical parameters including central tendency (e.g. means) or other basic estimates (e.g. regression coefficient) AND variation (e.g. standard deviation) or associated estimates of uncertainty (e.g. confidence intervals)
☐	☒	For null hypothesis testing, the test statistic (e.g. *F*, *t*, *r*) with confidence intervals, effect sizes, degrees of freedom and *P* value noted<br>*Give P values as exact values whenever suitable.*
☒	☐	For Bayesian analysis, information on the choice of priors and Markov chain Monte Carlo settings
☒	☐	For hierarchical and complex designs, identification of the appropriate level for tests and full reporting of outcomes
☐	☒	Estimates of effect sizes (e.g. Cohen's *d*, Pearson's *r*), indicating how they were calculated

Our web collection on statistics for biologists contains articles on many of the points above.

Software and code

Policy information about availability of computer code

Data collection	Binding Antibody Multiplex data were collected using a BioRad 200 machine and Bioplex Manager software, version 6.1, Security Edition. BLI assays were carried out using Fortebio OctetRed 384 instruments and biosensors (Fortebio- Biologics by Molecular Devices, San Jose, CA). Both data acquisition and analyses were performed with United States Food and Drug Administration's Title 21 Code of Federal Regulations Part 11 (FDA Title 21 CFR Part 11) compliant software versions (Data Acquisition 9.0 and Data Analysis 9.0/10.0 packages).
Data analysis	R statistical software (version 4.0.4)

For manuscripts utilizing custom algorithms or software that are central to the research but not yet described in published literature, software must be made available to editors and reviewers. We strongly encourage code deposition in a community repository (e.g. GitHub). See the Nature Research guidelines for submitting code & software for further information.

Data

Policy information about availability of data

All manuscripts must include a data availability statement. This statement should provide the following information, where applicable:

- Accession codes, unique identifiers, or web links for publicly available datasets
- A list of figures that have associated raw data
- A description of any restrictions on data availability

All data generated or analyzed during this study are included in this published article (and its supplementary information files) or in other publications described. All raw data are available upon request.

Field-specific reporting

Please select the one below that is the best fit for your research. If you are not sure, read the appropriate sections before making your selection.

☒ Life sciences ☐ Behavioural & social sciences ☐ Ecological, evolutionary & environmental sciences

For a reference copy of the document with all sections, see nature.com/documents/nr-reporting-summary-flat.pdf

Life sciences study design

All studies must disclose on these points even when the disclosure is negative.

Sample size	Sample sizes were chosen based on clinical trial design as described in the individual clinical trial protocols.
Data exclusions	We included all available data. Avidity index was only calculated for post-vaccination timepoints and positive responders. Any data imputation is described in the Methods section.
Replication	The Binding Antibody Multiplex Assay tested all samples in duplicate.<br>The BLI binding data tested all samples in triplicate.
Randomization	All clinical trials described in this manuscript were randomized Phase I clinical trials.
Blinding	Investigators were blinded to group allocation during data collection and analysis.

Reporting for specific materials, systems and methods

We require information from authors about some types of materials, experimental systems and methods used in many studies. Here, indicate whether each material, system or method listed is relevant to your study. If you are not sure if a list item applies to your research, read the appropriate section before selecting a response.

Materials & experimental systems

n/a	Involved in the study	
☐	☒	Antibodies
☒	☐	Eukaryotic cell lines
☒	☐	Palaeontology and archaeology
☒	☐	Animals and other organisms
☐	☒	Human research participants
☐	☒	Clinical data
☒	☐	Dual use research of concern

Methods

n/a	Involved in the study	
☒	☐	ChIP-seq
☒	☐	Flow cytometry
☒	☐	MRI-based neuroimaging

Antibodies

Antibodies used	The CSP C-terminal region–specific mAb AB236 and NANP repeat region–specific mAb AB334 were recombinantly produced as IgG1 and IgG3 mAbs (LakePharma, Belmont, CA and Duke Human Vaccine Institute Protein Production Facility, Durham, NC).
Validation	anti-human IgG1 (BioLegend, clone 12G8G11; https://www.biolegend.com/en-us/products/purified-anti-human-igg1-antibody-14306), anti-human IgG2 (Southern Biotech, clone HP6002; https://www.southernbiotech.com/techbul/9070.pdf), anti-human IgG3 (Invitrogen, clone HP6047; https://www.thermofisher.com/antibody/product/Mouse-anti-Human-IgG3-Heavy-chain-Secondary-Antibody-clone-HP6047-Monoclonal/05-3600), or anti-human IgG4 (BD Pharmingen, clone JDC-14; https://www.bdbiosciences.com/us/applications/research/b-cell-research/immunoglobulins/human/purified-mouse-anti-human-igg4-jdc-14/p/555878).

Human research participants

Policy information about studies involving human research participants

Population characteristics	Population characteristics for each clinical trial are described in the primary manuscript for each trial, referenced in the Results section, subheading "Controlled Human Malaria Infection Model (CHMI)".
Recruitment	Participants were recruited into each study protocol through local clinical sites.
Ethics oversight	The protocols were approved by the Walter Reed Army Institute of Research (WRAIR) Institutional Review Board and the Western Institutional Review Board. The trial was undertaken in accordance with International Council for Harmonisation of

Technical Requirements for Pharmaceuticals for Human Use guidelines and good clinical practice. Written informed consent was obtained from each subject before study procedures were initiated.

Note that full information on the approval of the study protocol must also be provided in the manuscript.

Clinical data

Policy information about clinical studies

All manuscripts should comply with the ICMJE guidelines for publication of clinical research and a completed CONSORT checklist must be included with all submissions.

Clinical trial registration	NCT01366534, NCT01857869
Study protocol	The full trial protocols may be accessed from GlaxoSmithKline upon request
Data collection	Clinical data and samples were collected according to the study protocol and at study sites as listed in clinicaltrials.gov. Binding antibody data were collected at Duke University according to the study protocol and assay specific study plans.
Outcomes	Data reported in this manuscript were exploratory for each study protocol. The primary and secondary outcomes of each trial are defined in the study protocol and listed on clinicaltrials.gov
